# The applicability of the “surprise question” as a prognostic tool in patients with severe chronic comorbidities in a university teaching outpatient setting

**DOI:** 10.1186/s12909-023-04714-2

**Published:** 2023-10-12

**Authors:** C. A. Lin, P. P. Pires, L. V. Freitas, P. V. S. Reis, F. D. Silva, L. G. Herbst, R. Nunes, C. J. Lin, M. P. T. Nunes

**Affiliations:** 1 Hospital das Clínicas da Faculdade de Medicina da Universidade de São Paulo, São Paulo, Brazil; 2 Hospital Sírio-Libanês, São Paulo, Brazil; 3 Universidade Do Porto, Porto, Portugal; 4 Universidade de São Paulo, São Paulo, Brazil

**Keywords:** Advanced directive, Palliative care, Surprise question, Outpatient, Noncommunicable chronical diseases

## Abstract

**Background:**

Life expectancy in recent decades has increased the prevalence of chronic diseases in the population, requiring an approach to new health topics, such as discussions on quality of life and expectations about death and dying. The concept of advance directives (ADs) gives individuals the opportunity to make known their decisions about the treatments they would like to receive at the end of life. Despite the recognition of relevance in clinical practice, the applicability of the concept presents challenges, including establishing the appropriate prognosis for each patient and the ideal time to approach the patient. Some prognostic tools were developed, such as the surprise question (SQ): *“Would you be surprised if your patient died in 12 months?”*, which is used in some clinical settings to predict patient deaths and to make decisions regarding ADs. The main objective of the present study was to evaluate the behavior of second-year resident physicians (PGY-2) when the SQ was applied.

**Method:**

In our observational study, from July 1, 2016, to February 28, 2017, (PGY-2) in the Internal Medicine Residency Program (IMRP) applied SQ to all patients with multiple and varied chronic no communicable comorbidities, who were followed up at the general medicine outpatient clinic (GMOC) of a tertiary university hospital in São Paulo- Brazil. The frequency of the outcome (death or non-death within 12 months) was analyzed by correlating it with the clinical data (impact of the studied variables).

**Results:**

Eight hundred forty patients entered the study. Fitfty-two of them (6.2%) died within one year. PGY-2 predicted that two hundred and fourteen patients (25.5% of total) would die within a year (answer No to SQ), of which, 32 (14.9%) did so. The correct residents’ prognosis for the subgroup of 626 patients (answer “Yes” to SQ) was NPV = 96.8% (CI = 95.4%-98.2%) and PPV = 14.9% (CI 10.1%-19, 6%). Answering “Yes” to SQ correlated negatively to addressing AD while the outcomes death and the answer No to SQ were positively correlated, according to the number of comorbidities.

**Conclusion:**

The SQ, in addition to care, contributed to health education, communication and care planning shared by the doctor and patient.

**Supplementary Information:**

The online version contains supplementary material available at 10.1186/s12909-023-04714-2.

## Background

Life expectancy in Brazil has increased in recent decades. Data from the Brazilian National Institute of Geography and Statistics (IBGE) indicate that the average life expectancy is 76.8 years (IBGE-DOU 25/11/2021). As a consequence, the prevalence of incurable and noncommunicable chronic diseases has increased in the population, making discussions about the quality of life and expectations about the process of death and dying essential.

Despite the aging of the Brazilian population and a large segment of the population living with chronic, progressive, and incurable diseases, proper end-of-life care planning is not being accomplished. Palliative care is in its infancy in Brazil and the delivery of optimal palliative and end-of-life is inconsistent across the country. Large numbers of individuals still die in the hospital and intensive care unit settings, without having discussions of end-of-life preferences and goals of care. Moreover, physicians lack knowledge and skills in basic principles of palliative care, including prognostication, goal setting and discussion of preferences of care at the end of life.

According to Dantas [1], Nowadays Medicine has to respond to *“two contradictory imperatives: to give the best possible care, but within mandatory limits and constraints* (..)”. *“A long time has passed from the days when physicians told patients what they needed and patients agreed without question.” *[1].

In other words, in Brazil, patients have the right to refuse any treatments to postpone the end of life with suffering, as long as they are fully informed about their clinical conditions, prognosis and expected results. Thus, the well-informed patient takes control of the decision-making process about end-of-life care. On the other hand, there should be respect to the patient´s autonomy and choice^3^.

At the end of the 1960s, the “living will” guidelines for ADs appeared in the United States as a way for patients to make end-of-life decisions about life support treatments and may include the choice of a legal representative to guarantee that their directives may be carried out if they lose the capacity to make their desires known [1–3].

AD has experienced recognition of its importance and has been widely discussed, resulting in specific laws and non-governmental movements around the world, such as “death with dignity”, which seek to expand the rights of patients to decide on their end of life care [4–7].

In Latin America, Puerto Rico was the first country to legislate ADs, and later, Argentina and Uruguay also did so [5]. In Brazil, the AD is still poorly understood and widespread. There are no specific legal acts about. Although the Brazilian Federal Constitution (Article 5, section VI and VIII) guarantees the competent patient´s autonomy to refuse treatment, after being properly informed, as well as 2002 Brazilian Civil Code, Elder Statute, Statute of Organ Donation and Transplants and some other State´s Health Laws did [1].

For this reason, on August 31, 2012, the Federal Council of Medicine (Conselho Federal de Medicina—CFM) approved Resolution CFM 1.995, which provides for ADs [8]. This document defines them as the set of desires, previously and expressly manifested by patients, about the care and treatment they want to receive or not if they are unable to freely express their will. If the patient has designated a representative for this purpose, the representative’s decisions will be taken into account by the health team, but the patient's desires will prevail over any other nonmedical opinion, including the families. Only decisions in disagreement with the code of medical ethics guarantee the professional the right to revoke them [8].

Regarding Palliative Care (PC) and public health policies, Resolution 41 was approved on October 31, 2018, which provides for the organization of PC within the Unified Health System (SUS) and regulates its provision at any level of care [9].

Hassegawa reviewed 22 studies with interviews and testimonies of physicians, intensivists and geriatricians, nurses, nursing technicians and assistants, medical students, lawyers and law students were conducted, as well as integrative and literature reviews. Some of the authors’ conclusion are that there is still a struggle evolving Brazilian society culture, individual values and technical criteria. In addition, the health team, if it has full control of the subject, fears carrying out the patient's will and suffering the consequences of the judicialization, given the lack of specific legal support. [10].

One of the challenges is to know the appropriate time to start the discussion about ADs with a patient [11, 12]. The literature shows that palliative treatment, initiated at the appropriate time, results in improved care, with lower costs for the individual and the health care system [3]. Understanding the patient, their values, their life and their family should precede the discussion about future priorities and care preferences. Planning end-of-life directives is a dynamic process. Studies show that patient preferences vary over time and that the choices about the proposed treatments depend on the health and life context. Billings and Bernacki point out that, generally, the proposal of ADs can occur very early, very late or at the right time. Commonly, patients decide for more "aggressive" "treatments when they are approached at the beginning of the course of their diseases^11.^ An approach that is too late tends to ignore the values, desires and preferences of the patient regarding end-of-life care, and often occurs in emergency rooms or intensive care units, initiated by young doctors, who often are still in professional training and in timely patient care [13].

The time to begin the palliative care approach depends on many factors, but an essential element is the evaluation of the prognosis of the disease. The patient needs to be aware of his or her diagnosis, prognosis and treatment options when facing life threating diseases; the health team needs to know and understand the values, goals and preferences of the patient. According to Kubler-Ross in her book “On death and dying”, the majority of patients know when they are facing to a terminally illness even when they have not been told. They stated that they would like to be told if they have a serious condition, but not with hope. This reinforce the importance of access the patients´ perceptions and values and to train the doctors how to prognosticate and emphatic communication [14].

The proposal of ADs needs to be made with appropriate time for the patient to be able to reflect and make the decisions, together with his or her family [13]. Therefore, trying to identify those who benefit most from this type of approach is essential [11, 13]. There are several ways to predict the progression of chronic patients; however, there is no method with 100% sensitivity and specificity [13]. Christakis [12] conducted a cohort study with 468 patients involving 343 physicians in reference “hospices”. The median survival of patients was 24 days. Approximately 20% of the prognoses were accurate, 63% were overly optimistic and 17% were pessimistic [12], which is why tools are used to assist in the difficult and imprecise task of prediction.

A useful method that can help identify patients at high risk of dying is the strategy of the surprise question (SQ), in which the doctor is asked to answer: *“Would you be surprised if your patient died in 12 months?* “The health professional answers “no” if the patient has a high chance of death in this period and “yes” in the opposite case. Thus, it is intended to reduce errors in the act of predicting [11, 15].

Cited for the first time in 2001, the surprise question was developed to direct patients to the program *“Improving Care through the End of Life”* in Washington. In this program, physicians responsible for primary care identified patients with severe and progressive diseases, and if they answered “no” to the surprise question, these patients were referred to palliative care specialists [16]. Since then, the surprise question has been used frequently [13, 16–18] in the USA. Studies have shown a sensitivity variation of 61.4% to 75% and specificity of 70% to 90.8% [16–18]. The method was initially studied to evaluate patients with cancer [11] or chronic kidney disease [17] and was also used as screening in general clinical practice to identify patients with indications for palliative care [19].

Brazil is a country of continental dimensions with clear and extreme social inequalities, in a predominantly paternalistic care model, with rare access to PC, advanced care planning (ACP) or AD. ACP conversations, if they occur at all, are usually late in the course of diseases. It is also associated with the lack of opportunity to educate health professionals about shared decision-making, which potentiates negative effects on vulnerable populations [20].

In Brazilian teaching hospitals, there is an increase in the prevalence of patients with more than one serious and incurable chronic diseases. However, the provision of care is, in most cases, still centered on the doctors, with the use of polypharmacy, without active participation of patients and relatives, despite the poor prognosis.

As a consequence to the lack of under graduation and physicians’ training in the provision of palliative and end-of-life care, prognostication and discussion of ADs are not routinely performed. Thus, there is an urgent need to broaden the healthcare team view and the care of patients preparing physicians, especially resident physicians, to address essential palliative care domains such as prognostication and ACP. Moreover, reliable prognostic assessment tools for use in clinical practice are lacking in Brazil.

The objective of this study was to validate the Surprise Question tool to Portuguese and to pilot it as a prognostic tool in an outpatient general medicine clinic at a large tertiary university hospital in Brazil. We also assessed whether the utilization of the SQ impacted the completion of AD during clinic visits.

## Methods

### Research design

This was an observational study (cohort) in which the main outcome was death and not death at 12 months. The tool, called the surprise question (SQ), was translated and validated for the Portuguese language, explained to resident physicians and fixed on computers in the care rooms: ‘Would you be surprised if your patient died in 12 months?’, with a simple, binary, “yes” or “no” response.

The translation and validation of SQ were performed by two translators native in Portuguese and fluent in English creating a consensus version. Then, the consensus version was applied to a group of physicians to evaluate their complete comprehension, as recommend by validation literature. Minor review of the SQ was applied. After which minor adjusts improved the SQ. A blind back translation was made by an external bilingual translator [21].

The study participants were 60 second year internal medicine residents (PGY-2) who participated in the outpatient general medicine clinic weekly during their residency training. Each resident provided longitudinal care to a panel of patients under the supervision of attending physicians. Prior to the study, residents attended a single and mandatory 60-min seminar on Advanced Directives. They also received didactic material twenty days before the seminar, including legislation from the Federal Council of Medicine and published papers on ADs. In addition, written questions were distributed to residents to potentially generate interest on the topic. Reading the material was voluntary.

The following topics were covered during the AD training session:

1. Chronic disease trajectory, prognostication and identifying patients appropriate for palliative care;
2. Ethical and legal aspects of ADs;
3. Writing an AD;
4. Basic communication skills on discussing AD with patients and relatives*, including concepts of* tolerable *truth (truth telling in health care*) and stages of grief.
5. Performing a basic spiritual assessment.

Once the training was completed, residents were instructed and encouraged to use the SQ during clinic visits.

Once for every patient, at the end of appointment, the residents answered the SQ, and if they addressed or not ADs. At the end of the day one of the investigators recorded all data in a separate database. (Diagram—Study design).

Data entry occurred from February 2016 to July 2017. After 12 months, outcome data (death or current living conditions) was obtained by chart review of phone contact with the patient/family. Patients demographic data (age, gender) and number of comorbidities was collected by chart review. Responses to the SQ and completion of an AD were also obtained by chart review.
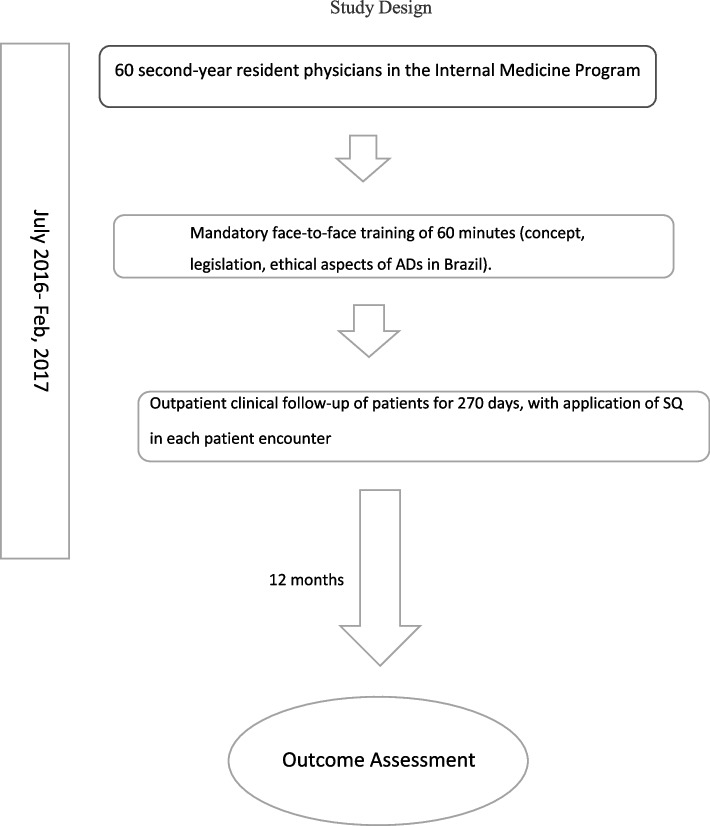


The study was approved by the Institutional Review Board. All resident physicians who agreed to participate signed an informed consent form.

### Statistical analysis

Frequency analysis was used to evaluate the number of associated comorbidities for each patient with positive and negative outcomes.

The quantitative data are described as the means, standard deviations and medians.

Continuous variables were defined as the mean ± standard deviation. Linear models were fitted to assess the impact of factors in determining outcomes.

The chi-square test was applied to analyze the relationship between death outcome and number of comorbidities; between the number of comorbidities and the response; between death outcome and answer to the surprise question; and between death versus response to the SQ and AD completion. As the number of comorbidities can be grouped into ordered categories, the relationship between the no response and the number of comorbidities was also evaluated using the Chi squared test for trend.

Logistic regression was used to analyze the relationship between each explanatory variable (age, sex, number of comorbidities) and the surprise question and the death and nondeath outcomes. 

Sensitivity, specificity, accuracy, and positive predictive values (PPV) and negative predictive values (NPV) for the SQ tool were calculated, as well as confidence intervals (CIs) for these values. To compare means, the nonparametric Wilcoxon test was used, and Fisher’s exact test was used for proportions. The CI is an interval that contains the true value of the measure with a probability of 95%.

The graphs are presented in histograms and boxplots constructed from the arrangement of the values, facilitating the visualization of how the data are distributed in four groups (quartiles) with the same number of samples. The second quartile is the value that divides the data into two groups with the same number of values. The box is drawn beginning in the 1st quartile and ending in the 3rd quartile and contains 50% of the most central data. The dash in the middle of the box is the median. Points marked with a circle or asterisk are values that can be considered extreme.

A level of significance of 5% was considered, which is equivalent to a CI of 95%. The statistical analyses were performed using JMP® Pro version 13—SAS Institute Inc., Cary, NC, USA, 1989–2019 and the freely available statistical language R (version 4.2.1, The Comprehensive R Archive Network).

## Results

**Fig. 1:**
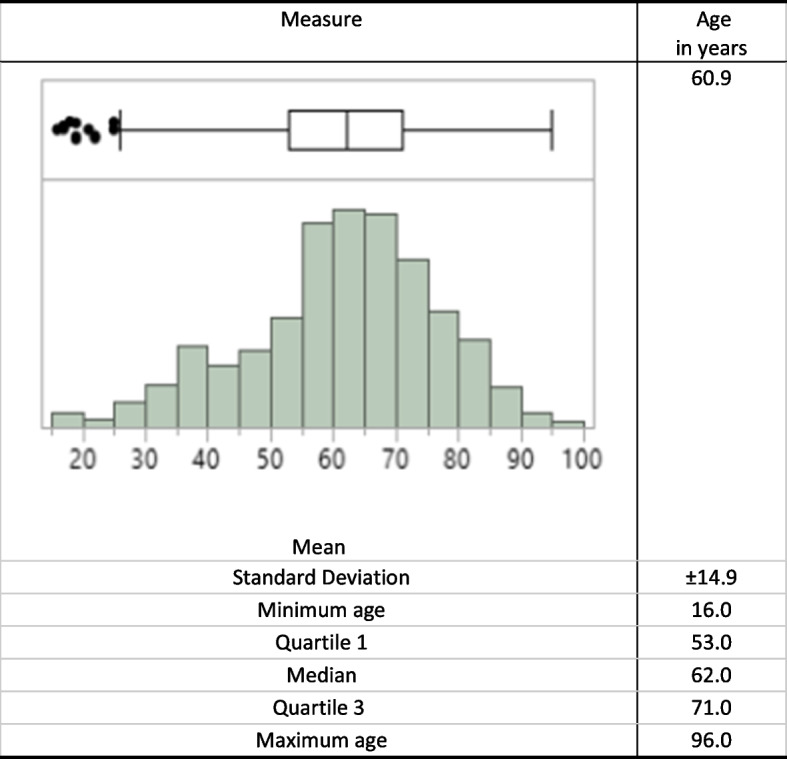
Depicts distribution of the patients by age group Age groups of the patients: minimum 16 years old, maximum 96 years old, mean 60.9 years old. The boxplot shows the 25th and 75th percentiles, with a median at the center. Quartile 1 = 53 years of age and quartile 3 = 75 years of age. The dark spots represent unusual values for the assisted population

A total of 840 patient encounters were recorded during the study period. Most patients were females (68.1%); the mean age was 60.9 years (SD ± 14.9 years); and the average number of comorbidities was 4.6 (SD ± 2.2). Figure 1 depicts the age distribution of the patients of the Outpatient Unit, where the resident physicians had their encounters.

Two hundred and fourteen patients (25.5%) had a “No” answer on the SQ. Of the 840 patients, 52 (6.2%) died within 12 months.

AD was completed in only 3.2% of the total (*n* = 27). Of these 27 patients, 21 were in the group to which the residents answered “No” to the SQ and only 6 patients were in the group to which the answer was “Yes”. (Tables 1 and 2).

**Table 1:** Resident physicians’ responses to the surprise question, outcomes (death or not) and approach to ADs

		**Participants %**	
		Absolute number	**% of** total sample
**Response of resident physicians to the surprise question**	No	214	25.5
	Yes	626	74.4
**Were Advance Directives addressed?**	No	813	96.8
	Yes	27	3.2
**Outcomes**	Non -death	788	93.8
	Death	52	6.2

Absolute numbers and percentages of resident physicians' responses to the surprise question; approach to advance directives and outcomes

**Table 2:** Shows the sensitivity and specificity values for the residents’ responses to the surprise question in regard to the outcome of death

Measure	Value	IC95%
Sensitivity	61.5%	48.3%-74.8%
Specificity	76.8%	73.9%-79.8%
Accuracy	75.9%	73.0%-78.8%
NPV	96.8%	95.4%-98.2%
PPV	14.9%	10.1%-19.6%

Sensitivity, specificity, accuracy, negative predictive value (NPV) and positive predictive value (PPV) value and their respective confidence intervals, considering the outcome death or non-death in one year

To refine the statistical analysis, the data were reanalyzed using logistic regression and Chi-square test as a tool. The results are presented below:

1. Number of comorbidities (up to 4 comorbidities X 5 or more comorbidities) and outcomes:
  a Progression to death was associated with the number of morbidities (Chi square = 5.1638,df = 1, *p* value = 0.02306).
  b The answer to the surprise question was associated with the number of morbidities of the patients (Chi square = 42.593, df = 1, *p* value = 6.741 × 10^-11).
  c The AD approach was associated with the number of comorbidities of the patients (Chi square = 16.21, df = 1, *p* value = 5.669 × 10^-05).
2. Analysis of the proportions (outcome-ordered categories of number of morbidities)
  - The evolution to death did not show a significant association with the increase in the number of morbidities (X-squared = 6.6104, df = 5, *p* value = 0.2513).
  - The answer to the surprise question showed a statistically significant association with the number of morbidities in the trend analysis (Chi square = 55.263, df = 5, *p* value = 1.153e-10).
  - The AD approach showed a statistically significant association with the number of morbidities in the trend analysis (X-squared = 19.144, df = 5, *p* value = 0.001807).
3. Relationship between progression to death and response to the surprise question and AD approach
  - The answer to the surprise question was associated with the evolution of the patient's death (X-squared = 35.706, df = 1, *p* value = 2.295e-09).
  - The approach to AD was not associated with the evolution of the patient's death (X-squared = 2.232, df = 1, *p* value = 0.1352).
4. Logistic regression
  - The probability of answering “yes” to the surprise question is not associated with the patient’s gender.
  - "The probability of answering" "yes" to the surprise question has an inverse association with the age of the patient (the older the patient is, the lower the probability of answering "yes").
  - The probability of answering “yes” to the surprise question is inversely and linearly associated with morbidity categories (the lower the number of morbidities is, the greater the probability of answering “yes”).
  - The association of the probability of answering “yes” to the surprise question with the number of morbidities does not change after adjusting for the age of the patients; and the probability of addressing ADs is not associated with the patient’s gender.
  - The probability of addressing ADs is associated with the patient’s age.
  - The probability of addressing ADs is associated with the fact that the patient has up to 4 (does not address) or 5 or more morbidities (addresses), but there is no linear relationship with the number of morbidities.
  - After adjusting for age, the probability of addressing ADs is no longer significantly associated with the fact that the patient belongs to the group with up to 4 morbidities or to the group that has 5 or more morbidities, i.e., the patient’s age is more important for the resident physician when addressing ADs than the number of comorbidities.
  - The probability of progression to death was not associated with the patient’s sex.
  - The probability of progression to death is significantly associated with the age of the patient.
  - The probability of progression to death is significantly associated with the fact that the patient has up to 4 (lower mortality) or more than 5 morbidities (higher mortality).
  - The association of the probability of progression to death with the number of morbidities of the patient disappeared after adjusting for the patient's age (i.e., the patient's age was a more important factor in determining death than the number of comorbidities).

## Discussion

This was the first study on the use of the Portuguese version of the Surprise Question as a prognostic tool in a general medicine teaching clinic at a tertiary teaching hospital in Brazil.

Young and yet inexperienced medical residents (PGY-2), were invited to apply the surprise question to patients under their care (*n* = 840), in a high complexity outpatient clinic of a tertiary teaching hospital. Those patients, with chronic degenerative clinical picture (> 4 comorbidities/patient), have a wide variety of diagnoses. The most prevalent conditions were hypertension, heart failure, diabetes, chronic obstructive pulmonary disease, and chronic renal failure. It is a challenging outpatient care scenario with clinically complex patients and physicians in training as internists.

Despite the variety of patients' diagnoses and the physician's professional inexperience, we found sensitivity, specificity and accuracy of the SQ similar to other studies, in which the same tool was used. It is important to note that most of previous studies included specific patient subgroups (cancer, chronic kidney disease, heart failure) and physicians with greater professional experience. [18, 19, 22].

We found that a higher number of comorbidities was associated with a higher probability of having a “No” answer on the SQ and a higher completion rate of ADs.

**Fig. 2:**
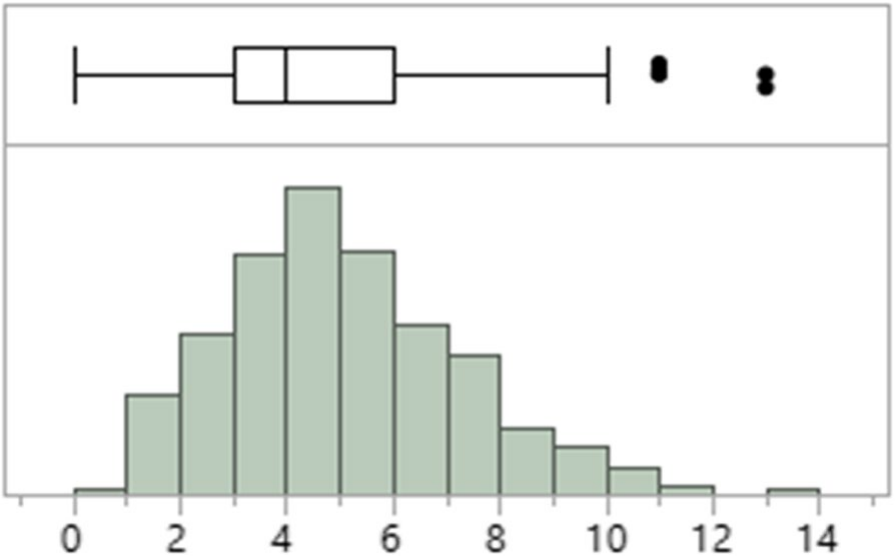
Depicts the distribution of the number of comorbidities in the patients. Boxplot of the distribution of comorbidities in the patients (*n* = 840). Quartile 1 = 3 comorbidities and quartile 3 = 6 comorbidities, with a median at the center. The dark dots represent unusual values for the assisted population

The higher number of comorbidities was significantly correlated with the lack of surprise on the part of residents regarding the death of their patients in one year, as well as providing a greater approach to ADs, although the patient's age was the main reason for this approach. There was no significant correlation between the number of comorbidities and the outcome of death (Table 3, Fig. 2).

**Table 3:** Shows the correlation between the number of comorbidities and the physicians’ response to the surprise question and whether, if positive, the ADs were addressed

		**Mean comorbidities/patient**	**Standard deviation** **( ±)**	***p* value**
**Response of Resident Physicians to the Surprise Question**	No	5.4	2.3	< 0.0001
	Yes	4.3	2.0	
**Were ADs addressed?**	No	4.5	2.2	< 0.0001
	Yes	6.1	1.5	
**Outcome**	Non -death	4.5	2.2	0.0799
	Death	5.1	2.3	

Mean comorbidities/patient, their association with physicians’ responses to the surprise question, ADs approach or not, outcome (death or not) with their respective standard deviations and p values

*ADs* advanced directives

There is still few published studies in terms of prognosis and advanced care plan for so complex and heterogeneous outpatients. We show that the greater the number of comorbidities per patient the higher is the association with addressing AD and to answer No to SQ. The patients' age is inversely proportional to the answer Yes to the SQ.

Despite the comorbidities profile of the patients, the number of deaths was low, which may have influenced the reduced value of PPV.

Our study also showed that resident physicians tend to choose survival more frequently than the death of their patients, as shown in Table 3. In a systematic review, Glare P et al.^15^ argued that although physicians consistently overestimate survival, their predictions are highly correlated with actual survival.

White et al., in a study with 50 vignettes (of which 14 dealt with cases of uncertain prognosis), concluded that, even when a patient had a more than 90% probability of death in 72 h, doctors made the correct estimate in 75% of the situations. The physician population in White’s study was consisted by volunteers with an average of more than 15 years of training and more than 10 years of practice in palliative care. The authors concluded that prognostication plays an important role in clinical practice; however, physicians should make clear to patients and family members the professional limitations of this practice [23].

In our study, resident physicians had less than three years of training and little or none experience in palliative care. Nevertheless, applying the surprise question, the NPV was higher than that obtained in the White study [23], where the participants had greater professional experience, except for the differences in the tools applied and the estimated time of death.

The surprise question favors the improvement of care because it allows the patient to be evaluated globally, rethink and plan treatment more cautiously, since physicians tend to overestimate a patient's prognosis [12].

According to data from the literature from 2003 to 2019, the PPV and NPV values of our study are consistent with the observation that physicians are better predictors of life than death [11, 17]. According to Martin, there are many reasons for that. Prognosis is not a routine part of clinical education and practice. Physicians are not well trained in how to estimate prognosis. Survival trials are biased, which limits their generalizability. Society's culture and discussions about death and dying favor professional norms that devalue prognostic estimates and favor optimism over accuracy. [19].

Another important fact in our study showed that, although we were not surprised by the possibility of death in one year, the proposal of advance directives was addressed to only 3,2% of all of the patients. According to our results, resident physicians tend to approach ADs with patients who have more comorbidities (more than 5) and of older ages. In our analyses, it was not possible to determine whether there was an age that precipitates the decision to approach ADs, but it was possible to distinguish the influence of the patient's age and the number of comorbidities above 5 as determinants for the residents to address ADs. When refining the statistical analysis, we found greater weight for age than for the number of comorbidities. The resident physician decided to approach ADs for polymorbid and older patients.

The present study demonstrates the need for greater effort in the training of physicians on approaching patients about ADs. In line with the literature, there is a need to develop a prognostic score for most patients. In addition, there is an urgent need to involve our society in the subject.

In the literature review, during the preparation of this manuscript, we found that the introduction to ADs applied to residents who participated in the present study occurred coincidentally according to the proposal of training physicians in conversations related to clinical conditions with poor prognosis, published after our study was completed [24].

In a systematic review with 16 studies, Glare et al. [25] sought to evaluate the prognostic properties of the SQ that were better in studies involving cancer patients than those with other diseases. In studies involving patients with noncancerous diseases, the likelihood ratios were less useful (LR set positive = 2.7 [95% CI 2.1–3.6] and negative LR = 0.53 [95% CI 0.46–0.61]). Heterogeneity was low or absent for the odds ratio and likelihood ratios but generally high for sensitivity, specificity, PPV and NPV for both cancer patients and other conditions. In contrast to our study, in this systematic review, there were 2/3 false-positives for patients with diseases other than cancer.

According to Glare, SQ was not originally conceived as a prognostic tool but rather as a screening test for patients who could benefit from a palliative approach. Since then, it has been used for palliative or end-of-life interventions and has even been incorporated as a screening and palliative needs test (NECPAL). According to the present study, clinical experience contributes to the performance of the surprise question, with moderate interobserver harmony [25].

According to the authors, further studies are needed to determine whether SP combined with other clinical indicators improves the identification of patients with palliative care needs [23].

Our study developed on the use of the SQ in a complex internal medicine outpatient setting in Brazil. It contributes strongly to strengthening the surprise question as a prognostic tool, especially for the introduction of talk about advance directives, in countries such as Brazil, where such strategies are not yet widely employed and the most paternalistic care prevails.

As in the present case, the SQ tool assists in the development of health care and education methods, improving professional training through prognostic communication strategies and care planning shared by the doctor and patient, in a clear expansion of the autonomy of the latter.

Although we have not studied it, it is clear that the lack of education in shared decision-making and palliative care is the most important barriers to PC, AD and ACP in Brazil, in the twenty-first century. It is urgent to change quickly in terms of health education, from undergraduation to practice in all of the Brazilian Health System levels.

### Limitations of this study

Limitations of our study included a relatively small sample of residents (60/1600) from a large academic institution, limiting the generalizability of our findings.

Furthermore, data entry took place for only 7 months.

The answer to the surprise question was based on data reported by the residente who previously treated the patient, without recording his/her impression of the patients´ prognosis.

Residents followed the patients for just under a year.

There was a time gap, longer than expected and for reasons beyond the control of the authors, between data collection and manuscript submission, although the literature still shows the relevance of our data.

## Conclusions

We found the implementation of the Portuguese version of the SQ was feasible in an internal outpatient setting. There is a higher level of sensitivity, specificity and accuracy in the sample of patients with chronic medical conditions. Predictors of a ‘No” answer on the SQ included the number of comorbidities and the age of the patient.

Physicians addressed advance directives for only 3.2% of patients who would not be surprised in case of death in a year. The AD application rate was low, reflecting the lack of knowledge on the subject by health professionals, including physicians. Three studies from 3 Brazilian medical schools showed that 6% to 24% of students understand the meaning of AD. The lack of knowledge/information about AD/ACP also involves the general population.

It is necessary to train all health professionals in activity and include Palliative Care as a mandatory subject in all health courses and residencies. Population enlightenment is fundamental.

Residents were five times more correct about the death prognosis for patients to whom they answered No to SQ. Survival was better predicted for the patients to whom residents answered YES to the SQ, NPV and PPV reinforce a lot of evidences showing doctors as good predictors of life. We need to increase the skill of prognosticate death since this skill implies a series of decision-making by the healthcare team, patients and surrogates.

The SQ has been successfully employed in a complex outpatient setting by resident physicians. SQ served as a tool to accelerate the understanding of each patients´ prognosis while simultaneously introducing the concept of palliative care and advance directives. Residents benefited most from the introduction to the aforementioned concepts. The low offer of AD disadvantaged patients and family members.

### Next steps

Continue the study in the outpatient clinic, reaching a greater number of resident physicians, as an education strategy in Palliative Care, introducing the concept of Advance Care Planning to the health team and to patients and families.

By receiving junior doctors from most Brazilian regions, it will be possible to assess whether the subject of palliative care has actually become more taught and assimilated by the new generations of doctors, as recent decision by the National Council of Education. In November 2022, the Brazilian National Council of Education updated the curricular guidelines for medical graduation in the country, including palliative care in the body of knowledge and skills that must be taught and acquired [26].

Repeat the study with the nursing and physiotherapist team in the Internal Medicine Ward. We shall contribute to their professional advancement in CP and ACP. As the nursing team spends more time with patients and has intense interaction with all health professionals, in that scenario, they will contribute a lot to the dissemination of concepts, acting as opinion makers.

Develop and evaluate the Entrustable Professional Activities related to ACP, AD and CP of physicians who are in progress and who had completed the Internal Medicine residency program in our institution.

### Supplementary Information

**Additional file 1: Supplementary file.** Table with date of patients included in the study, including gender, diagnosis, answer to the surprise question and outcome (death or non death)

## Data Availability

The authors declare that the raw data (some of them in Portuguese) are available and uploaded as supplement file.
