# Supplementary material for: The applicability of the “surprise question” as a prognostic tool in patients with severe chronic comorbidities in a university teaching outpatient setting
Source: BMC Med Educ. 2023 Oct 12;23:761. doi: 10.1186/s12909-023-04714-2 (PMC10571481; doi:10.1186/s12909-023-04714-2)
Supplement: Supplementary file 1 — Additional file 1: Supplementary file. Table with date of patients included in the study, including gender, diagnosis, answer to the surprise question and outcome (death or non death) [file 12909_2023_4714_MOESM1_ESM.docx]

Supplementary File

Table with date of patients included in the study, including gender, diagnosis, answer to the surprise question and outcome (death or non death).

| **Ages** | **Register number** | **Gender** | **Diagnosis** | **Answer to surprise question** | **Are de Advanced Directives apprached** | **outcome** |
| --- | --- | --- | --- | --- | --- | --- |
| 76 | 14176041h | Male | Hypertension, Diabetes Mellitus, Hypothyroidism, Chronic Pancreatitis | No | No | Non death |
| 62 | 14155206b | Male | Diabetes Mellitus, Dyslipidemia, Cirrhosis | No | Yes | Non death |
| 82 | 13968008e | Female | Hypertension, Hypothyroidism, Osteoarthritis , Rheumatoid Arthritis, Aortic Stenosis | No | No | Death |
| 79 | 14042627g | Female | Hypertension, Diabetes Mellitus, Dyslipidemia, Hypothyroidism | No | No | Non death |
| 81 | 2644284e | Female | Hypertension, Diabetes Mellitus, Hypothyroidism, Coronary Artery Disease (Symptomatic or Positive Testing) | No | No | Non death |
| 75 | 14049371h | Male | Chronic Kidney Disease, Coronary Artery Disease (Symptomatic or Positive Testing), Heart Failure, Chronic Obstructive Pulmonary Disease (COPD, emphysema), Atrial Fibrillation, Peripheral Artery Disease, | No | No | Death |
| 89 | 14171967g | Male | Diabetes Mellitus, Chronic Kidney Disease, Osteoarthritis , Anemia (with previous prescription of medical treatment or transfusion) | No | No | Non death |
| 57 | 14111795k | Female | Hypertension, Diabetes Mellitus, Chronic Kidney Disease, Heart Failure, Anemia (with previous prescription of medical treatment or transfusion), Pulmonary Hypertension, | No | No | Death |
| 72 | 60001640c | Male | Hypertension, Heart Failure, Cancer, Aortic Aneurysm | No | No | Non death |
| 78 | 14165084d | Male | Hypertension, Cancer, Cirrhosis | No | No | Non death |
| 69 | 13565455i | Female | Hypertension, Diabetes Mellitus, Dyslipidemia, Coronary Artery Disease (Symptomatic or Positive Testing), Chronic Obstructive Pulmonary Disease (COPD, emphysema), Depression | No | No | Non death |
| 56 | 14169736b | Male | Hypertension, Cancer | No | No | Death |
| 39 | 14155868b | Female | Cancer, Anemia (with previous prescription of medical treatment or transfusion), Bronchiectasis, Immunodeficiency | No | No | Non death |
| 63 | 13742112e | Female | Diabetes Mellitus, Chronic Obstructive Pulmonary Disease (COPD, emphysema), Smoker, Subclavian Artery Stenosis | No | No | Non death |
| 95 | 14130871j | Female | Hypertension, Heart Failure, Cancer, Anemia (with previous prescription of medical treatment or transfusion) | No | Yes | Death |
| 70 | 2218555e | Female | Hypertension, Dyslipidemia, Hypothyroidism, Chronic Kidney Disease, Coronary Artery Disease (Symptomatic or Positive Testing), Heart Failure, Osteoarthritis , CNS Aneurysm | No | No | Non death |
| 58 | 90834157a | Male | Hypertension, Chronic Kidney Disease, Heart Failure, Alcohol Use Disorder, Prediabetes | No | No | Non death |
| 78 | 13718420b | Female | Hypertension, Diabetes Mellitus, Hypothyroidism, Chronic Kidney Disease,Peripheral Artery Disease, Glaucoma, Cataract | No | No | Non death |
| 80 | 13932577b | Male | Hypertension, Diabetes Mellitus, Dyslipidemia, Coronary Artery Disease (Symptomatic or Positive Testing), Heart Failure, Chronic Obstructive Pulmonary Disease (COPD, emphysema), Smoker, Radiculopathy, Diabetic Amyotrophy | No | No | Non death |
| 64 | 14157040e | Female | Hypertension, Chronic Kidney Disease, Coronary Artery Disease (Symptomatic or Positive Testing), Previous Stroke or TIA, Previous Smoker, Carotid Artery Stenosis, Renal Artery Stenosis | No | No | Death |
| 84 | 5212566g | Female | Hypertension, Diabetes Mellitus, Dyslipidemia, Hypothyroidism, Coronary Artery Disease (Symptomatic or Positive Testing), Heart Failure, Osteoporosis, Dementia | No | Yes | Non death |
| 58 | 33322327g | Female | Diabetes Mellitus, Hypothyroidism, Cataract, Leiomyomas, Osteopenia | No | No | Non death |
| 81 | 2806453g | Female | Hypertension, Diabetes Mellitus, Dyslipidemia, Cancer, Chronic Lymphoid Leukemia, Chronic Low Back Pain, Diverticulosis, Dementia | No | No | Non death |
| 78 | 13835743j | Female | Hypertension, Diabetes Mellitus, Dyslipidemia, Chronic Kidney Disease, Coronary Artery Disease (Symptomatic or Positive Testing), Osteoarthritis , Previous Stroke or TIA, Gout, Benign Prostatic Hyperplasia | No | No | Non death |
| 83 | 14053940g | Male | Hypertension, Diabetes Mellitus, Chronic Kidney Disease, Coronary Artery Disease (Symptomatic or Positive Testing), Chronic Obstructive Pulmonary Disease (COPD, emphysema), Cancer, Mitral Regurgitation, Atrial Fibrillation, Chronic Venous Disease, Benign Prostatic Hyperplasia, | No | No | Non death |
| 88 | 13550993j | Female | Hypertension, Diabetes Mellitus, Osteoporosis, Onychomycosis, Cataract, Chronic Constipation, Depression, Peripheral Arterial Disease | No | No | Non death |
| 59 | 13770815j | Male | Hypertension, Diabetes Mellitus, Chronic Kidney Disease, Chronic Obstructive Pulmonary Disease (COPD, emphysema), Smoker, Chronic Pancitopenia, Depression | No | No | Non death |
| 85 | 13947809k | Male | Hypertension, Dyslipidemia, Hypothyroidism, Previous Smoker, Previous Alcohol Use Disorder, Disacusia,Cirrhosis, Atrial Fibrillation, Anxiety Disorder, Cholelithiasis, | No | No | Death |
| 75 | 2947786b | Female | Diabetes Mellitus, Dyslipidemia, Hypothyroidism, Coronary Artery Disease (Symptomatic or Positive Testing), Heart Failure, Osteoarthritis , Gastroesophageal Reflux, Depression, Diverticulosis | No | No | Non death |
| 60 | 90981540f | Male | Cirrhosis | No | No | Death |
| 64 | 14144110i | Male | Dyslipidemia, Chronic Obstructive Pulmonary Disease (COPD, emphysema), Osteoarthritis , Smoker | No | No | Non death |
| 46 | 13525056d | Female | Hypertension, Diabetes Mellitus, Systemic Lupus,Peripheral Artery Disease, Migraine,HPV | No | No | Non death |
| 69 | 14047971e | Female | Hypertension, Diabetes Mellitus, Rheumatoid Arthritis, Chronic Low Back Pain, Cardiomyopathy , Chagas Disease, | No | No | Non death |
| 92 | 13986371j | Female | Hypertension, Chronic Kidney Disease, Heart Failure, Osteoporosis, Arrythmia, Pulmonary Embolism, Deep Venous Thrombosis, Peptic Ulcer, Osteopenia, | No | Yes | Non death |
| 74 | 13968925j | Male | Hypertension, Diabetes Mellitus, Dyslipidemia, Chronic Kidney Disease, Chronic Obstructive Pulmonary Disease (COPD, emphysema), Cancer, Obesity, Previous Smoker | No | No | Non death |
| 63 | 4036151j | Male | Hypertension, Previous Stroke or TIA, Anemia (with previous prescription of medical treatment or transfusion), Previous Smoker, Previous Alcohol Use Disorder, Aortic Aneurysm, Polyarthritis, Atrial Fibrillation, | No | No | Non death |
| 72 | 3362287b | Male | Chronic Obstructive Pulmonary Disease (COPD, emphysema), Osteoporosis, Rheumatoid Arthritis | No | No | Death |
| 77 | 3312079h | Female | Hypertension, Diabetes Mellitus, Dyslipidemia, Previous Stroke or TIA | No | No | Non death |
| 63 | 3164464g | Male | Hypertension, Diabetes Mellitus, Dyslipidemia, Chronic Kidney Disease, Heart Failure, Chronic Obstructive Pulmonary Disease (COPD, emphysema), Peripheral Artery Disease, | No | No | Non death |
| 34 | 13730788c | Male | Chronic Kidney Disease, Systemic Lupus, Anxiety Disorder generalizada, Peptic Ulcer Disease , Hipertrigliceridemia, Hyperthyroidism, Hyperparathyroidism, | No | No | Non death |
| 77 | 14068216g | Female | Hypertension, Heart Failure, Osteoporosis, Chronic Low Back Pain, Atrial Fibrillation, Systemic Sclerosis, | No | Yes | Non death |
| 47 | 14034190i | Female | Hypertension, Diabetes Mellitus, Hypothyroidism, Chronic Kidney Disease, Heart Failure, Chronic Obstructive Pulmonary Disease (COPD, emphysema), Gastritis, Chronic Pancreatitis | No | No | Non death |
| 81 | 2340474h | Male | Hypertension, Cancer, Thyroid Nodule, Arthritis, Osteoarthritis, Benign Prostatic Hyperplasia, Glaucoma | No | No | Non death |
| 60 | 13970005i | Male | Hypertension, Chronic Obstructive Pulmonary Disease (COPD, emphysema) | No | No | Non death |
| 61 | 77085280d | Male | Diabetes Mellitus, Dyslipidemia, Obesity, Panic Disorder, Hypoacusia, | No | No | Non death |
| 72 | 55701521k | Female | Hypertension, Diabetes Mellitus, Dyslipidemia, Obesity | No | No | Non death |
| 66 | 5355131b | Male | Diabetes Mellitus, Coronary Artery Disease (Symptomatic or Positive Testing), Heart Failure, Chronic Obstructive Pulmonary Disease (COPD, emphysema), Obesity, | No | No | Death |
| 61 | 13986545g | Female | Osteoporosis, Toxic Multinodular Goiter, Deep Venous Thrombosis, | No | No | Non death |
| 66 | 13744007h | Male | Hypertension, Osteoarthritis , Esophagitis, Benign Prostatic Hyperplasia, Polyarthritis | No | No | Non death |
| 62 | 14138970k | Female | Hypertension, Diabetes Mellitus, Chronic Kidney Disease, Gout, Obesity, Obstructive Sleep Apnea | No | Yes | Non death |
| 65 | 13775857d | Female | Hypertension, Diabetes Mellitus, Chronic Kidney Disease, Coronary Artery Disease (Symptomatic or Positive Testing), Heart Failure, Obesity | No | Yes | Non death |
| 81 | 2699515b | Male | Hypertension, Diabetes Mellitus, Dyslipidemia, Chronic Kidney Disease, Coronary Artery Disease (Symptomatic or Positive Testing), Previous Stroke or TIA, Cancer | No | No | Non death |
| 82 | 3087243b | Female | Hypertension, Hypothyroidism, Heart Failure, Previous Stroke or TIA, Anemia (with previous prescription of medical treatment or transfusion), Dementia, | No | Yes | Non death |
| 55 | 4043391j | Male | Hypertension, Diabetes Mellitus, Dyslipidemia, Cirrhosis, Chronic Pancreatitis | Yes | No | Non death |
| 55 | 55527975c | Male | Coronary Artery Disease (Symptomatic or Positive Testing), Cancer, Pulmonary Embolism | Yes | No | Non death |
| 45 | 13985486c | Female | Systemic Lupus | Yes | No | Non death |
| 56 | 13953546f | Male | Hypertension, Diabetes Mellitus, Chronic Kidney Disease | Yes | No | Non death |
| 57 | 13750643a | Female | Hypertension, Diabetes Mellitus, Coronary Artery Disease (Symptomatic or Positive Testing), Systemic Lupus | Yes | No | Non death |
| 65 | 13772396f | Female | Hypertension, Diabetes Mellitus, Dyslipidemia | Yes | No | Death |
| 52 | 14095514e | Female | Hypertension, Diabetes Mellitus | Yes | No | Non death |
| 72 | 13925212f | Female | Hypertension, Dyslipidemia, Chronic Kidney Disease, Osteoarthritis , Systemic Lupus, Previous Stroke or TIA, inibidor adquirido de fator VIII; | Yes | No | Non death |
| 53 | 3249954c | Female | Rheumatoid Arthritis | Yes | No | Non death |
| 76 | 14083342k | Male | Hypothyroidism, Smoker, Benign Prostatic Hyperplasia, | Yes | No | Non death |
| 43 | 13661890j | Male | Hypertension, Diabetes Mellitus, Dyslipidemia | Yes | No | Non death |
| 35 | 90579017k | Female | Pulmonary Embolism | Yes | No | Non death |
| 72 | 2437584i | Male | Hypertension, Diabetes Mellitus, Dyslipidemia, Hypothyroidism, Chronic Hepatitis C | Yes | No | Non death |
| 35 | 13442451c | Female | Osteoporosis, Systemic Lupus | Yes | No | Non death |
| 75 | 14143206g | Male | Hypertension, Benign Prostatic Hyperplasia, Chronic Diarrhea, Iliac Artery Aneurysm | Yes | No | Non death |
| 57 | 13905157e | Female | Rheumatoid Arthritis | Yes | No | Non death |
| 35 | 13731845i | Female | Systemic Lupus | Yes | No | Non death |
| 66 | 3501066k | Male | Hypertension, Diabetes Mellitus, Dyslipidemia, Kidney Transplantion | Yes | No | Non death |
| 69 | 90982466d | Female | Hypertension, Chronic Obstructive Pulmonary Disease (COPD, emphysema), Pulmonary Embolism | Yes | No | Non death |
| 53 | 13495517d | Female | Diabetes Mellitus, Asthma | Yes | No | Non death |
| 69 | 13479200g | Female | Hypertension, Diabetes Mellitus, Dyslipidemia, Hypothyroidism | Yes | No | Non death |
| 62 | 14070667i | Female | Hypertension, Diabetes Mellitus, Dyslipidemia, Osteoporosis | Yes | No | Non death |
| 67 | 3330024d | Female | Hypertension, Diabetes Mellitus, Osteoporosis, Rheumatoid Arthritis | Yes | No | Non death |
| 74 | 90921840h | Female | Osteoporosis, Cirrhosis hepatica; Alcohol Use Disorder | Yes | No | Non death |
| 38 | 13871325d | Male | Hypertension, Diabetes Mellitus | Yes | No | Non death |
| 56 | 14138628h | Male | Diabetes Mellitus, Chronic Kidney Disease, Heart Failure | Yes | No | Non death |
| 69 | 13992955b | Male | Hypothyroidism, Coronary Artery Disease (Symptomatic or Positive Testing), Heart Failure, Chronic Obstructive Pulmonary Disease (COPD, emphysema), Atrial Fibrillation, | Yes | No | Non death |
| 68 | 3020125d | Female | Hypertension, Osteoarthritis , Asthma, Depression | Yes | No | Non death |
| 70 | 13995854c | Female | Hypertension, Diabetes Mellitus, Dyslipidemia, Depression | Yes | No | Non death |
| 51 | 7005464b | Female | Hypertension, Dyslipidemia , Hypothyroidism, Chronic Kidney Disease, Osteoporosis, Bipolar disorder | Yes | No | Non death |
| 74 | 13612362g | Female | Hypertension, Diabetes Mellitus, Dyslipidemia, Osteoarthritis , Osteoporosis, Depression | Yes | No | Non death |
| 70 | 3386351c | Female | Hypertension, Diabetes Mellitus, Dyslipidemia, Hypothyroidism, Chronic Kidney Disease | Yes | No | Non death |
| 45 | 13518505b | Female | Systemic Lupus, Gastroesophageal Reflux, Nephrolithiasis | Yes | No | Non death |
| 59 | 14037950g | Female | Hypertension, Chronic Kidney Disease, Osteoarthritis , Osteoporosis, Anemia (with previous prescription of medical treatment or transfusion), Gout, Gastroesophageal Reflux, Hyperparathyroidism, Depression | Yes | No | Non death |
| 50 | 13991315j | Male | Diabetes Mellitus, Dyslipidemia, Chronic Obstructive Pulmonary Disease (COPD, emphysema), Smoker, Cirrhosis | Yes | No | Non death |
| 49 | 14071329d | Female | Chronic Kidney Disease, Anemia (with previous prescription of medical treatment or transfusion), Urinary Tract Congenital Malformation | Yes | No | Non death |
| 49 | 14023502g | Female | Hypertension, Diabetes Mellitus, Hypothyroidism, Depression | Yes | No | Non death |
| 33 | 13976151h | Female | Osteoarthritis , Rheumatoid Arthritis | Yes | No | Non death |
| 83 | 3171608k | Male | Hypertension, Diabetes Mellitus, Coronary Artery Disease (Symptomatic or Positive Testing), Heart Failure | Yes | No | Non death |
| 39 | 3273847h | Female | Asthma, Systemic Lupus | Yes | No | Non death |
| 50 | 2876574i | Female | Osteoarthritis , Systemic Lupus | Yes | No | Non death |
| 71 | 55372459a | Male | Hypertension, Dyslipidemia, Coronary Artery Disease (Symptomatic or Positive Testing), Heart Failure, Osteoarthritis , Atrial Fibrillation, Chronic Pulmonary Embolism | Yes | No | Non death |
| 54 | 14069472b | Female | Hypothyroidism, Rheumatoid Arthritis | Yes | No | Non death |
| 88 | 13436565k | Male | Hypertension, Dyslipidemia, Hypothyroidism, Chronic Kidney Disease, Heart Failure, Osteoporosis | Yes | Yes | Non death |
| 66 | 13457271g | Female | Hypertension, Diabetes Mellitus, Chronic Kidney Disease, Osteoporosis, Rheumatoid Arthritis, Anemia (with previous prescription of medical treatment or transfusion), Depression | Yes | No | Non death |
| 82 | 13689420f | Male | Hypertension, Diabetes Mellitus, Dyslipidemia, Chronic Kidney Disease, Osteoarthritis , Gout; Benign Prostatic Hyperplasia | Yes | No | Non death |
| 52 | 13765211h | Male | Hypertension, Dyslipidemia, Heart Failure, Obstructive Sleep Apnea; Atrial Fibrillation | Yes | No | Non death |
| 67 | 13888397f | Female | Hypertension, Coronary Artery Disease (Symptomatic or Positive Testing), Heart Failure, Depression | Yes | No | Non death |
| 65 | 55531232d | Female | Hypertension, Coronary Artery Disease (Symptomatic or Positive Testing), Heart Failure, Chronic Obstructive Pulmonary Disease (COPD, emphysema), Smoker | Yes | No | Non death |
| 55 | 2171082c | Female | Dyslipidemia, Hypothyroidism, Osteoarthritis , Smoker | Yes | No | Non death |
| 65 | 13849063f | Female | Diabetes Mellitus, Dyslipidemia, Osteoporosis | Yes | No | Non death |
| 66 | 14156052e | Male | Hypertension, Diabetes Mellitus, Previous Stroke or TIA, Neurogenic Bladder | Yes | No | Death |
| 59 | 7010752c | Female | Chronic Obstructive Pulmonary Disease (COPD, emphysema), Osteoporosis, Smoker, Bipolar Disorder | Yes | No | Non death |
| 55 | 2460000h | Female | Hypertension, Rheumatoid Arthritis, Smoker, Depression | Yes | No | Non death |
| 63 | 14142632b | Male | Hypertension, Generalized Edema | Yes | No | Non death |
| 61 | 13844012c | Male | Hypertension, Heart Failure | Yes | No | Non death |
| 38 | 14153894g | Male | Hypertension, Hypothyroidism, Depression | Yes | No | Non death |
| 51 | 14177067b | Male |  | Yes | No | Non death |
| 58 | 13553600d | Female | Hypertension, Diabetes Mellitus, Asthma | Yes | No | Non death |
| 47 | 3219906a | Male | Hypertension, Diabetes Mellitus, Dyslipidemia, Depression | Yes | No | Non death |
| 61 | 55385623i | Female | Hypertension, Diabetes Mellitus, Rheumatoid Arthritis, Mitral Valve disease | Yes | No | Non death |
| 74 | 2001906c | Female | Hypertension, Diabetes Mellitus, Dyslipidemia, Hypothyroidism, Chronic Kidney Disease, Osteoarthritis , Anemia (with previous prescription of medical treatment or transfusion) | Yes | No | Non death |
| 64 | 2923339g | Male | Sarcoidosis; Chronic Subdural Hematoma | Yes | No | Non death |
| 68 | 3324375f | Female | Hypertension, Diabetes Mellitus, Dyslipidemia, Depression | Yes | No | Non death |
| 57 | 3322206g | Female | Hypertension, Diabetes Mellitus, Dyslipidemia, Asthma, Urinary Incontinence | Yes | No | Non death |
| 75 | 2627888g | Male | Hypertension, Diabetes Mellitus, Dyslipidemia, Chronic Kidney Disease, Coronary Artery Disease (Symptomatic or Positive Testing), Gout | Yes | No | Non death |
| 70 | 3284626a | Male | Hypertension, Diabetes Mellitus, Hypothyroidism, Chronic Kidney Disease | Yes | No | Non death |
| 28 | 90842214e | Female | Hypertension, Systemic Lupus | Yes | No | Non death |
| 70 | 13991719a | Female | Hypertension, Diabetes Mellitus, Dyslipidemia, Chronic Kidney Disease, Heart Failure, Osteoarthritis | Yes | No | Non death |
| 76 | 3281576e | Male | Hypertension, Diabetes Mellitus, Dyslipidemia, Chronic Kidney Disease | Yes | No | Non death |
| 65 | 90782068j | Male | Hypertension, Dyslipidemia, Coronary Artery Disease (Symptomatic or Positive Testing), Heart Failure, Previous Stroke or TIA | Yes | No | Non death |
| 72 | 14139307k | Male | Hypertension, Dyslipidemia, Atrial Fibrillation, Graves Disease | Yes | No | Non death |
| 61 | 2825391e | Male | Hypertension, Diabetes Mellitus, Dyslipidemia, Atrial Fibrillation, Nephrolithiasis, Chronic Venous Disease | Yes | No | Non death |
| 43 | 14055852g | Female | Fibromyalgia , hepatopatia crônica a/e, Doença de still do adulto, anemia ferropriva, Leiomyomas, | Yes, No | No | Non death |
| 65 | 13714938b | Female | Hypertension, Diabetes Mellitus, Chronic Kidney Disease, Coronary Artery Disease (Symptomatic or Positive Testing), Osteoporosis, Colonic Polyps, Chronic Cholecystitis, Synovial Cysts | Yes | No | Non death |
| 19 | 14123004g | Female | Hypertension, Diabetes Mellitus, Dyslipidemia, Rheumatoid Arthritis | Yes | No | Non death |
| 61 | 90556670c | Female | Heart Failure, Chronic Obstructive Pulmonary Disease (COPD, emphysema), Primary Hyperparathyroidism | Yes | No | Non death |
| 36 | 14078491g | Female | Dyslipidemia, Rheumatoid Arthritis, Dyspepsia, Intolerancia a lactose, Smoker, Obesity, Vitamin D Deficiency | Yes | No | Non death |
| 75 | 13714564b | Female | Hypertension, Diabetes Mellitus, Dyslipidemia, Hypothyroidism, Coronary Artery Disease (Symptomatic or Positive Testing), Heart Failure, Osteoarthritis , Cancer, Osteopenia | Yes | No | Non death |
| 60 | 3158441b | Female | Hypertension, Diabetes Mellitus, Dyslipidemia, Osteoporosis, Obesity, Intermittent Claudication | Yes | No | Non death |
| 75 | 3319918k | Female | Hypertension, Dyslipidemia, Hypothyroidism, Osteoporosis, Graves Optic Neuropathy + Cataract + Glaucoma, Chronic Venous Insufficiency, Allergic Rhinitis | Yes | No | Non death |
| 78 | 13550536h | Female | Hypertension, Diabetes Mellitus, Cancer, nontoxic multinodular goiter, Osteopenia | Yes | No | Non death |
| 68 | 13497193a | Female | Hypertension, Diabetes Mellitus, Dyslipidemia, Osteoporosis, Thyroid NoduSystemic Lupus , Depression, Statin-induced Autoimmune Necrotizing Myopathy, | Yes | No | Non death |
| 45 | 13536359k | Female | Hypothyroidism, Rheumatoid Arthritis, Polycystic Ovary Syndrome , Allergic Rhinitis, Parkinsonismo secundário? , Epilepsy, BPreviousiga neurogênica, | Yes | No | Non death |
| 71 | 90882933c | Male | Diabetes Mellitus, Dyslipidemia, Previous Smoker, Previous Alcohol Use Disorder, Pulmonary Tuberculosis tratada, B Hepatitis curada, CANDIDIASE ESOFÁGICA | Yes | No | Non death |
| 62 | 14050024e | Female | Dyslipidemia, Osteoarthritis , trombofilia a/e, Obesity Prediabetes, | Yes | No | Non death |
| 65 | 14121386a | Male | Smoker, Hyperthyroidism, Pre Diabetes, Obesity | Yes | No | Non death |
| 62 | 14131018d | Female | Rheumatoid Arthritis, Previous Smoker | Yes | No | Non death |
| 74 | 14147881j | Female | Hypertension, Asthma, Rheumatoid Arthritis, Previous Stroke or TIA | Yes | No | Non death |
| 82 | 3136745h | Male | Hypertension, Diabetes Mellitus, Hypothyroidism, Coronary Artery Disease (Symptomatic or Positive Testing), Osteoarthritis , Previous Smoker | Yes | No | Non death |
| 19 | 14168972b | Female | Systemic Lupus | Yes | No | Non death |
| 85 | 2362866d | Male | Hypertension, Dyslipidemia, Hypothyroidism, Chronic Kidney Disease, Previous Stroke or TIA, Talassemia beta-minor , Peripheral Artery Disease, Pré- DM, Previous Alcohol Use Disorder e Previous Smoker, | Yes | No | Non death |
| 30 | 13560063e | Female | Hypothyroidism, Systemic Lupus, Migraine, Osteopenia, Sickle Cell trait , Non erosive Deforming Arthritis | Yes | No | Non death |
| 59 | 14121883f | Female | Hypertension, Asthma, Systemic Lupus, Sjogren Disease, Chronic Tension-type Headache, Gastroesophageal Reflux, Fibromyalgia , Relapsing Polychondritis | Yes | No | Non death |
| 38 | 14056187e | Male | Rheumatoid Arthritis | Yes | No | Non death |
| 37 | 13941877k | Male | Diabetes Mellitus, Hypothyroidism, Asthma, Onychomycosis | Yes | No | Non death |
| 74 | 3372300a | Female | Hypertension, Diabetes Mellitus, Dyslipidemia, Cirrhosis por NASH, Osteopenia, Hematoma subdural crônico | Yes | No | Non death |
| 58 | 14103900i | Male | Hypertension, Diabetes Mellitus, Chronic Kidney Disease, Coronary Artery Disease (Symptomatic or Positive Testing), Heart Failure,Left Ventricular Thrombus | Yes | No | Non death |
| 62 | 13510421k | Female | Hypertension, Dyslipidemia, Osteoporosis, Rheumatoid Arthritis | Yes | No | Non death |
| 75 | 13807507a | Male | Hypertension | Yes | No | Non death |
| 82 | 13771848d | Female | Hypertension, Dyslipidemia, Hypothyroidism | Yes | No | Non death |
| 51 | 13552377d | Female | Hypertension, Systemic Lupus, Anemia (with previous prescription of medical treatment or transfusion), Depression | Yes | No | Non death |
| 38 | 14165191k | Female | Systemic Sclerosis | Yes | No | Non death |
| 46 | 13708919d | Female | Hypertension, Diabetes Mellitus, Dyslipidemia, Hypothyroidism | Yes | No | Non death |
| 34 | 13439622g | Female | Hypertension, Diabetes Mellitus | Yes | No | Non death |
| 76 | 13951642e | Male | Hypertension, Diabetes Mellitus, Dyslipidemia, Chronic Kidney Disease, Coronary Artery Disease (Symptomatic or Positive Testing), Heart Failure, Depression; Parkinson | Yes | No | Non death |
| 56 | 13473560g | Female | Hypertension, Diabetes Mellitus, Dyslipidemia, Osteoarthritis , Previous Stroke or TIA, Depression | Yes | No | Non death |
| 78 | 13437560k | Female | Hypertension, Diabetes Mellitus, Dyslipidemia, Chronic Kidney Disease, Chronic Obstructive Pulmonary Disease (COPD, emphysema), Previous Stroke or TIA | Yes | No | Non death |
| 50 | 13745627f | Female | Hypertension, Dyslipidemia, Rheumatoid Arthritis | Yes | No | Non death |
| 42 | 13801899a | Male | Hypothyroidism, Osteoporosis, Kidney and Pancreas Transplantion | Yes | No | Non death |
| 68 | 13695855a | Female | Hypertension, Osteoarthritis , Rheumatoid Arthritis | Yes | No | Non death |
| 37 | 13908643j | Female | Deep Vein Thrombosis | Yes | No | Non death |
| 65 | 14040388j | Female | Diabetes Mellitus | Yes | No | Non death |
| 56 | 2857629k | Female | Hypertension, Diabetes Mellitus, Hypothyroidism, Asthma, Depression | Yes | No | Non death |
| 68 | 3308985d | Female | Hypertension, Diabetes Mellitus, Dyslipidemia, Osteoarthritis , Depression | Yes | No | Non death |
| 76 | 55432155a | Male | Hypertension, Diabetes Mellitus, Dyslipidemia, Chronic Kidney Disease, Coronary Artery Disease (Symptomatic or Positive Testing), Chronic Obstructive Pulmonary Disease (COPD, emphysema), Dementia, Peripheral Artery Obstruction | Yes | No | Non death |
| 57 | 13528029f | Male | Hypertension, Dyslipidemia, Hypothyroidism, Chronic Kidney Disease, Coronary Artery Disease (Symptomatic or Positive Testing), Heart Failure | Yes | No | Non death |
| 72 | 3019651f | Female | Hypertension, Coronary Artery Disease (Symptomatic or Positive Testing), Rheumatoid Arthritis | Yes | No | Non death |
| 64 | 2130258e | Female | Hypertension, Diabetes Mellitus, Dyslipidemia, Depression | Yes | No | Non death |
| 75 | 5020202d | Male | Hypertension, Diabetes Mellitus, Dyslipidemia, Chronic Kidney Disease, Atrial Fibrillation | Yes | No | Non death |
| 78 | 14006626a | Male | Hypertension, Chronic Kidney Disease, Osteoporosis | Yes | No | Non death |
| 74 | 4003692j | Female | Hypertension, Diabetes Mellitus, Dyslipidemia, Chronic Kidney Disease, Heart Failure, Rheumatoid Arthritis | Yes | No | Non death |
| 50 | 13879813d | Female | Hypertension, Systemic Lupus, Smoker | Yes | No | Non death |
| 33 | 13822174a | Female | Chronic Kidney Disease, Systemic Lupus | Yes | No | Non death |
| 49 | 14156679a | Female | Hypertension, Diabetes Mellitus, Dyslipidemia, Hypothyroidism, Depression | Yes | No | Non death |
| 58 | 13652726h | Female | Hypertension, Rheumatoid Arthritis | Yes | No | Non death |
| 59 | 90657433h | Female | Hypertension, Heart Failure, Anemia (with previous prescription of medical treatment or transfusion), Hereditary Telangectasia | Yes | No | Non death |
| 66 | 13594212h | Female | Dyslipidemia, Hypothyroidism, Rheumatoid Arthritis | Yes | No | Non death |
| 58 | 90816035d | Male | Hypertension, Diabetes Mellitus, Dyslipidemia, Hypothyroidism, Obesity, Hyperuricemia e Gouty Arthritis relatada, Hepatic Steatosis, Benign Prostatic Hyperplasia sintomática, Gastroesophageal Reflux, Sd. Depressivo-ansiosa em tratamento, Previous Smoker | Yes | No | Non death |
| 73 | 13543181e | Female | Hypertension, Diabetes Mellitus, Dyslipidemia, Chronic Kidney Disease, transtorno depressivo controlado, Obesity Grau II | Yes | No | Non death |
| 61 | 89503069e | Female | Hypertension, Diabetes Mellitus, Dyslipidemia, Osteoarthritis , Obesity, Síndrome do tunel do carpo. | Yes | No | Non death |
| 39 | 13826290h | Female | Rheumatoid Arthritis, Glaucoma, Obesity | Yes | No | Non death |
| 73 | 5040994a | Female | Hypertension, Dyslipidemia, Hypothyroidism, Coronary Artery Disease (Symptomatic or Positive Testing), Osteoarthritis , Osteoporosis, Depression/Anxiety Disorder, Cataract, Glaucoma, Functional Dyspepsia., | Yes | No | Non death |
| 72 | 13981108b | Female | Hypertension, Diabetes Mellitus, Osteoarthritis , Ulcerative Colitis, Obesity, Benign paroxysmal positional vertigo | Yes | No | Non death |
| 51 | 33396233e | Male | Hypothyroidism, Anemia (with previous prescription of medical treatment or transfusion), Hypothyroidism Miopatia , Psychiatric Disorders with Auditory Hallucinations | Yes | No | Non death |
| 64 | 55376838e | Female | Hypertension, Diabetes Mellitus, Dyslipidemia, Heart Failure, Depression, Chronic Low Back Pain | Yes | No | Non death |
| 87 | 3358022i | Male | Hypertension, Dyslipidemia, Chronic Obstructive Pulmonary Disease (COPD, emphysema), Cataract, Depression, Pre Diabetes mellitusa, aortic stenosis | Yes | No | Non death |
| 61 | 60016525j | Female | Hypertension, Diabetes Mellitus, Cancer, Obesity, Previous Smoker, Osteopenia | Yes | No | Non death |
| 37 | 14035813h | Female | Systemic Lupus | Yes | No | Non death |
| 78 | 13858353g | Male | Hypertension, Hypothyroidism, Chronic Kidney Disease, Coronary Artery Disease (Symptomatic or Positive Testing), Heart Failure, Benign Prostatic Hyperplasia, | Yes | No | Non death |
| 64 | 13514709j | Female | Hypertension, Diabetes Mellitus, Dyslipidemia, Hypothyroidism, Osteoarthritis , Previous Stroke or TIA | Yes | No | Non death |
| 35 | 13954399b | Female | Systemic Lupus | Yes | No | Non death |
| 51 | 3078814i | Female | Hypertension, Hypothyroidism, Systemic Lupus | Yes | No | Non death |
| 70 | 2497980e | Female | Hypertension, Diabetes Mellitus, Hypothyroidism | Yes | No | Non death |
| 60 | 14043162d | Male | Dyslipidemia, Hypothyroidism, Chronic Pancreatitis | Yes | No | Non death |
| 67 | 14139138i | Female | Hypertension, Diabetes Mellitus, Heart Failure, Osteoarthritis | Yes | No | Non death |
| 74 | 2947786b | Female | Diabetes Mellitus, Dyslipidemia, Hypothyroidism, Coronary Artery Disease (Symptomatic or Positive Testing), Heart Failure, Osteoarthritis , Depression | Yes | No | Non death |
| 66 | 2407888k | Female | Hypertension, Diabetes Mellitus, Dyslipidemia, Asthma | Yes | No | Non death |
| 44 | 3163304a | Female | Hypertension, Diabetes Mellitus, Dyslipidemia, Hypothyroidism, Chronic Kidney Disease | Yes | No | Non death |
| 72 | 55705266h | Male | Hypertension, Coronary Artery Disease (Symptomatic or Positive Testing) | Yes | No | Non death |
| 45 | 14158562e | Female | Dyslipidemia, Hypothyroidism, Anemia (with previous prescription of medical treatment or transfusion) | Yes | No | Non death |
| 38 | 13860399a | Female | Hypertension, Hypothyroidism | Yes | No | Non death |
| 29 | 14006431k | Male | Hypothyroidism | Yes | No | Non death |
| 44 | 14040110c | Female | Hypertension, Dyslipidemia, Hypothyroidism, psoriasis vulugar | Yes | No | Non death |
| 73 | 2055431b | Female | Hypertension, Diabetes Mellitus, Dyslipidemia, Smoker | Yes | No | Death |
| 31 | 13860977g | Female | Systemic Lupus | Yes | No | Non death |
| 62 | 3138515d | Female | Hypertension, Diabetes Mellitus, Asthma, Depression | Yes | No | Non death |
| 32 | 14163348j | Female | Deep Venous Thrombosis | Yes | No | Non death |
| 69 | 13994338h | Female | Hypertension, Diabetes Mellitus, Atrial Fibrillation;Pressure Ulcer | Yes | No | Death |
| 60 | 3235448d | Female | Hypertension, Dyslipidemia, Rheumatoid Arthritis | Yes | No | Non death |
| 51 | 3266555j | Female | Hypertension, Diabetes Mellitus, Dyslipidemia, Smoker | Yes | No | Non death |
| 28 | 14102272h | Female | Diabetes Mellitus | Yes | No | Non death |
| 75 | 14079874k | Male | Hypertension, Hypothyroidism, Chronic Kidney Disease, Smoker | Yes | No | Death |
| 56 | 14074767g | Male | Hypertension, Chronic Kidney Disease, Chronic Obstructive Pulmonary Disease (COPD, emphysema) | Yes | No | Non death |
| 51 | 14140242c | Male | Hypertension, Diabetes Mellitus, Cancer | Yes | No | Non death |
| 52 | 14140200f | Female | Hypertension, Diabetes Mellitus, Dyslipidemia, Depression | Yes | No | Non death |
| 78 | 13962983b | Female | Hypertension, Diabetes Mellitus, Dyslipidemia, Heart Failure, Atrial Fibrillation; | Yes | No | Non death |
| 77 | 90638072i | Female | Hypertension, Diabetes Mellitus, Dyslipidemia,Aortic Stenosis; | Yes | No | Non death |
| 67 | 13784180g | Female | Hypertension, Diabetes Mellitus, Dyslipidemia, Hypothyroidism, Asthma, Depression | Yes | No | Non death |
| 71 | 13668518C | Male | Hypertension, Diabetes Mellitus, Dyslipidemia, Chronic Kidney Disease, Anemia (with previous prescription of medical treatment or transfusion),Low Members Amputation, Diabetic Retinopathy/Diabetic Neuropathy, Benign Prostatic Hyperplasia, Chronic Wound in Lower Member | No | No | Non death |
| 45 | 13435832E | Female | Systemic Lupus, Síndrome de sjogren, Furuncurosis | No | No | Non death |
| 68 | 14006878I | Female | Hypertension, Diabetes Mellitus, Dyslipidemia, Hypothyroidism, Coronary Artery Disease (Symptomatic or Positive Testing), Anemia (with previous prescription of medical treatment or transfusion), otosclerosis,Erosive Pangastritis, Benign paroxysmal positional vertigo, B Hepatitis | No | No | Non death |
| 61 | 13505909C | Male | Asthma, Depression, CIA, abdominal wal hernia | No | No | Non death |
| 47 | 14034190I | Female | Hypertension, Diabetes Mellitus, Hypothyroidism, Chronic Kidney Disease, Heart Failure, Chronic Obstructive Pulmonary Disease (COPD, emphysema), Sliding Hiatus HerniaErosive Gastritis, Chronic Pancreatitis | No | No | Non death |
| 81 | 3247475D | Female | Hypertension, Diabetes Mellitus, Dyslipidemia, Osteoarthritis , Previous Stroke or TIA, Dementia | No | No | Non death |
| 58 | 2729625H | Female | Hypertension, Diabetes Mellitus, Dyslipidemia, Anemia (with previous prescription of medical treatment or transfusion), Irritable Bowel Syndrome / Lactose Intolerance, Diverticulosis, Epilepsy, Hepatic Steatosis, Depression | No | No | Non death |
| 71 | 13799226F | Male | Hypertension, Diabetes Mellitus, Chronic Kidney Disease, Meig Sd., Onychomycosis, Tinea Pedis | No | No | Non death |
| 70 | 2078565B | Female | Hypertension, Diabetes Mellitus, Dyslipidemia, Chronic Kidney Disease, Breast Cancer , Cryoglobulinemia, Interstitial Pneumonia | No | No | Non death |
| 71 | 13955110E | Female | Hypertension, Diabetes Mellitus, Dyslipidemia, Rheumatoid Arthritis, Carpal Tunnel Syndrome , Low Back Pain, Dyspepsia | No | No | Non death |
| 79 | 14001161F | Female | Hypertension, Diabetes Mellitus, Dyslipidemia, Restrictive Lung Disease with Hypoxemia, Incisional Hernia, Depression | No | No | Non death |
| 76 | 2121021H | Female | Hypertension, Dyslipidemia, Coronary Artery Disease (Symptomatic or Positive Testing), Osteoporosis, Epilepsy | No | No | Non death |
| 76 | 2338556G | Female | Hypertension, Diabetes Mellitus, Cancer with Metastasis | No | No | Non death |
| 38 | 13822673D | Male | Systemic Lupus | No | No | Non death |
| 72 | 13554411C | Male | Hypertension, Diabetes Mellitus, Dyslipidemia, Hypothyroidism, Coronary Artery Disease (Symptomatic or Positive Testing), Asthma, Breast Cancer | No | No | Non death |
| 72 | 13554411C | Female | Hypertension, Diabetes Mellitus, Dyslipidemia, Hypothyroidism, Coronary Artery Disease (Symptomatic or Positive Testing), Asthma | No | No | Non death |
| 38 | 14155868B | Female | Cancer, Anemia (with previous prescription of medical treatment or transfusion), Bronchiectasis | No | No | Non death |
| 68 | 13740926I | Male | Hypertension, Bronchiectasis | No | No | Non death |
| 72 | 2685499D | Female | Hypertension, Diabetes Mellitus, Dyslipidemia, Hypothyroidism, Chronic Kidney Disease | No | No | Non death |
| 64 | 3108924D | Male | Hypertension, Diabetes Mellitus, Chronic Kidney Disease, Previous Stroke or TIA | No | No | Non death |
| 65 | 14098153F | Female | Hypertension, Diabetes Mellitus, Pulmonary Embolism | No | No | Non death |
| 63 | 14079586G | Male | Diabetes Mellitus, Cirrhosis | No | No | Non death |
| 61 | 3212432G | Female | Hypertension, Hypothyroidism, Chronic Obstructive Pulmonary Disease (COPD, emphysema), Systemic Lupus | No | No | Non death |
| 61 | 3181676H | Female | Hypertension, Diabetes Mellitus, Dyslipidemia, Hypothyroidism, Heart Failure, Chronic Obstructive Pulmonary Disease (COPD, emphysema) | No | Yes | Non death |
| 70 | 13524796B | Female | Cirrhosis | No | No | Non death |
| 66 | 5363585D | Female | Hypertension, Diabetes Mellitus, Dyslipidemia, Hypothyroidism, Chronic Kidney Disease, Heart Failure, Previous Stroke or TIA | No | No | Non death |
| 92 | 3341513J | Female | Hypertension, Diabetes Mellitus, Dyslipidemia, Chronic Kidney Disease, Osteoporosis, Dementia, IGg Monoclonal Peak | No | No | Non death |
| 51 | 14164006I | Male | Hypertension, Diabetes Mellitus, Coronary Artery Disease (Symptomatic or Positive Testing),Depression, Cushing Syndrome | No | No | Non death |
| 81 | 3363437D | Male | Hypertension, Dyslipidemia, Chronic Kidney Disease, Heart Failure, Hyperuricemia , Prediabetes, Atrial Fibrillation, Ascending Colon Angioectasy, Diverticulosis , Cancer em 1994 | No | No | Non death |
| 91 | 2409489E | Male | Hypertension, Diabetes Mellitus, Chronic Kidney Disease, Chronic Obstructive Pulmonary Disease (COPD, emphysema), Arrhythmia, Previous Smoker | No | Yes | Non death |
| 75 | 3359245I | Female | Hypertension, Dyslipidemia, Hypothyroidism, Osteoarthritis , Low Back Pain (Hip Osteoarthritis) , Depression/Anxiety Disorder, Gastroesophageal Reflux, Allergic Rhinitis, Diverticulosis colônica, ReObsessive-compulsive Disorderele grau II, | No | No | Non death |
| 38 | 14179073D | Female | Gastritis, Fibromyalgia | No | No | Non death |
| 86 | 14095354A | Female | Hypertension, Dyslipidemia, Heart Failure, Osteoporosis, Dementia, Prediabetes | No | Yes | Non death |
| 70 | 2031777D | Female | Hypertension, Diabetes Mellitus, Dyslipidemia, Hypothyroidism, Asthma, Previous Stroke or TIA, Depression/ Anxiety Disorder,Peripheral Venous Insufficiency | No | Yes | Non death |
| 67 | 14112122J | Female | Hypertension, Diabetes Mellitus, Dyslipidemia, Chronic Obstructive Pulmonary Disease (COPD, emphysema), Previous Smoker, hypovitaminose D, Rhinitis | No | No | Non death |
| 53 | 14121705J | Female | Dyslipidemia, Hypothyroidism, Hyperthyroidism - Graves Disease | No | No | Non death |
| 70 | 13550657H | Male | Hypertension, Diabetes Mellitus, Dyslipidemia, Hypothyroidism, Chronic Kidney Disease, Previous Stroke or TIA, Previous Smoker, Glaucoma, Paroxysmal Atrial Fibrillation? | No | No | Non death |
| 68 | 13550657H | Male | Hypertension, Dyslipidemia, Hypothyroidism, Chronic Kidney Disease, Previous Stroke or TIA | No | No | Non death |
| 91 | 2475260F | Female | Hypertension, Dyslipidemia, Hypothyroidism, Chronic Kidney Disease, Heart Failure, Osteoarthritis , Osteoporosis, Dementia, Cancer | No | No | Non death |
| 83 | 77078369J | Male | Hypertension, Diabetes Mellitus, Dyslipidemia, Chronic Kidney Disease, Dementia | No | No | Non death |
| 65 | 14127382H | Male | Hypertension, Diabetes Mellitus, Hypothyroidism, Chronic Kidney Disease, Obesity | No | No | Non death |
| 90 | 13969340F | Female | Hypertension, Dyslipidemia, Hypothyroidism, Previous Stroke or TIA, Dementia | No | Yes | Non death |
| 63 | 5149231I | Male | Hypertension, Diabetes Mellitus, Hypothyroidism, Chronic Kidney Disease, Coronary Artery Disease (Symptomatic or Positive Testing), Heart Failure | No | No | Non death |
| 60 | 14149135F | Male | Hypertension, Diabetes Mellitus, Chronic Kidney Disease, Coronary Artery Disease (Symptomatic or Positive Testing) | No | No | Non death |
| 59 | 3113885H | Female | Hypertension, Diabetes Mellitus, Dyslipidemia, Hypothyroidism, Osteoarthritis , Obesity, Depression | No | No | Non death |
| 71 | 14092811K | Female | Hypertension, Hypothyroidism, Chronic Obstructive Pulmonary Disease (COPD, emphysema), Previous Stroke or TIA, Cancer | No | No | Non death |
| 59 | 13974482G | Female | Hypertension, Diabetes Mellitus, Dyslipidemia, Previous Stroke or TIA | No | No | Non death |
| 83 | 3201386C | Male | Hypertension, Dyslipidemia, Osteoporosis | No | No | Non death |
| 59 | 2504461D | Female | Hypertension, Diabetes Mellitus, Osteoarthritis , Osteoporosis, Rheumatoid Arthritis | No | No | Non death |
| 50 | 3172237C | Female | Systemic Lupus, Anemia (with previous prescription of medical treatment or transfusion), Antiphospholipid Syndrome | No | No | Non death |
| 66 | 13765705I | Male | Hypertension, Diabetes Mellitus, Dyslipidemia | No | No | Non death |
| 61 | 14138970K | Female | Hypertension, Diabetes Mellitus, Chronic Kidney Disease, Obstructive Sleep Apnea | No | Yes | Non death |
| 43 | 13938863K | Female | Asthma, Gastroesophageal Reflux | No | No | Non death |
| 51 | 14153083J | Female | Hypertension, Dermatomyositis | No | No | Non death |
| 76 | 14093327F | Female | Hypertension, Diabetes Mellitus, Chronic Kidney Disease, Heart Failure, Osteoarthritis , Atrial Fibrillation | No | No | Non death |
| 54 | 14034983J | Female | Hypertension, Diabetes Mellitus, Dyslipidemia, Osteoarthritis | No | No | Non death |
| 74 | 3095640D | Female | Hypertension, Diabetes Mellitus, Dyslipidemia, Hypothyroidism, Heart Failure, Chronic Obstructive Pulmonary Disease (COPD, emphysema), Deep Vein Thrombosis | No | No | Non death |
| 76 | 5367604E | Female | Diabetes Mellitus, Coronary Artery Disease (Symptomatic or Positive Testing), Heart Failure, Dementia, Cancer | No | No | Non death |
| 72 | 14120751D | Female | Hypertension, Dyslipidemia, Hypothyroidism, Chronic Kidney Disease, Chronic Obstructive Pulmonary Disease (COPD, emphysema) | No | Yes | Non death |
| 76 | 14176041H | Male | Hypertension, Diabetes Mellitus, Hypothyroidism, Anemia (with previous prescription of medical treatment or transfusion), Chronic Pancreatitis, | No | No | Non death |
| 73 | 3219558K | Female | Hypertension, Diabetes Mellitus, Osteoarthritis , Osteoporosis, Rheumatoid Arthritis, Pulmonary Tuberculosis; Paroxysmal Atrial Tachycardia | No | No | Non death |
| 61 | 13980158C | Female | Hypertension, Diabetes Mellitus, Smoker, Peripheral Artery Disease | No | No | Non death |
| 83 | 14127491B | Male | Hypertension, Diabetes Mellitus, Dyslipidemia, Chronic Kidney Disease, Coronary Artery Disease (Symptomatic or Positive Testing), Heart Failure | No | Yes | Non death |
| 86 | 13641527D | Female | Hypertension, Heart Failure, Osteoarthritis , Osteoporosis, Megacolon due to Chagas Disease | No | No | Non death |
| 75 | 3321444E | Female | Hypertension, Diabetes Mellitus, Dyslipidemia, Previous Stroke or TIA, Dementia | No | No | Non death |
| 56 | 13923343B | Female | Hypertension, Diabetes Mellitus, Dyslipidemia, Hypothyroidism, Cancer, Obstructive Sleep Apnea; Depression | No | No | Non death |
| 80 | 55396676B | Male | Hypertension, Diabetes Mellitus, Dyslipidemia, Hypothyroidism, Chronic Kidney Disease, Heart Failure, Chronic Obstructive Pulmonary Disease (COPD, emphysema), Benign Prostatic Hyperplasia, Gout | No | No | Non death |
| 34 | 77075948H | Female | Hypertension, Hypothyroidism, Chronic Kidney Disease, Bipolar Disorder | No | No | Non death |
| 58 | 13675171F | Female | Hypertension, Dyslipidemia, Osteoporosis, Systemic Lupus, Cancer, Deep Venous Thrombosis; | No | No | Non death |
| 55 | 13991218K | Female | Hypertension, Diabetes Mellitus, Dyslipidemia, Hypothyroidism, Smoker | No | No | Non death |
| 63 | 13866107H | Female | Hypertension, Diabetes Mellitus, Dyslipidemia, Chronic Obstructive Pulmonary Disease (COPD, emphysema), Osteoarthritis , Obstructive Sleep Apnea, | No | No | Non death |
| 51 | 14162526D | Female | Hypertension, Diabetes Mellitus, Chronic Kidney Disease, Coronary Artery Disease (Symptomatic or Positive Testing), Heart Failure | No | No | Non death |
| 67 | 55534208C | Female | Chronic Obstructive Pulmonary Disease (COPD, emphysema), Smoker | No | No | Non death |
| 70 | 55326704H | Male | Hypertension, Diabetes Mellitus, Dyslipidemia, Hypothyroidism, Chronic Kidney Disease | No | No | Non death |
| 58 | 13905419C | Female | Diabetes Mellitus, Hypothyroidism | No | No | Non death |
| 62 | 13679744A | Female | Hypertension, Diabetes Mellitus, Dyslipidemia, Hypothyroidism, Coronary Artery Disease (Symptomatic or Positive Testing), Chronic Obstructive Pulmonary Disease (COPD, emphysema), Depression | No | No | Non death |
| 72 | 88242335E | Female | Hypertension, Diabetes Mellitus, Dyslipidemia, Chronic Obstructive Pulmonary Disease (COPD, emphysema), Previous Stroke or TIA | No | No | Non death |
| 84 | 13805119K | Male | Hypertension, Diabetes Mellitus, Chronic Kidney Disease, Coronary Artery Disease (Symptomatic or Positive Testing), Heart Failure, Chronic Obstructive Pulmonary Disease (COPD, emphysema), Previous Stroke or TIA, Dementia, Abdominal Aortic Aneurysm | No | No | Non death |
| 65 | 14008358C | Female | Dyslipidemia, Osteoarthritis , Púrpura de Henoch-Schonlein; Depression | No | No | Non death |
| 83 | 13701697G | Female | Hypertension, Diabetes Mellitus, Dyslipidemia, Osteoarthritis | No | No | Non death |
| 69 | 5270965J | Female | Hypertension, Diabetes Mellitus, Dyslipidemia, Osteoarthritis | No | No | Non death |
| 55 | 44206100H | Female | Systemic Lupus | No | No | Non death |
| 82 | 13970638F | Male | Hypertension, Diabetes Mellitus, Dyslipidemia, Chronic Kidney Disease, Heart Failure, Osteoporosis, Rheumatoid Arthritis, Cancer | No | No | Non death |
| 59 | 14006755K | Female | Hypertension, Diabetes Mellitus, Dyslipidemia | No | No | Non death |
| 72 | 13462499D |  | Hypertension, Chronic Obstructive Pulmonary Disease (COPD, emphysema), Osteoporosis, Systemic Lupus | No | No | Non death |
| 56 | 14070868K | Female | Hypertension, Hypothyroidism, Coronary Artery Disease (Symptomatic or Positive Testing), Osteoarthritis | No | No | Non death |
| 43 | 77117469G | Female | Systemic Lupus, Fibromyalgia | No | No | Non death |
| 63 | 2908105D | Female | Hypertension, Diabetes Mellitus, Epilepsy | No | No | Non death |
| 76 | 3322557A | Female | Hypertension, Diabetes Mellitus, Dyslipidemia, Hypothyroidism, Coronary Artery Disease (Symptomatic or Positive Testing) | No | No | Non death |
| 57 | 90962481D | Female | Chronic Obstructive Pulmonary Disease (COPD, emphysema) | No | No | Non death |
| 45 | 13485173A | Female | Hypertension, Diabetes Mellitus, Cancer, Depression | No | No | Non death |
| 72 | 14139307K | Male | Hypertension, Dyslipidemia, Atrial Fibrillation; Hyperthyroidism | No | No | Non death |
| 71 | 3218445D | Male | Hypertension, Diabetes Mellitus, Dyslipidemia, Chronic Kidney Disease, Coronary Artery Disease (Symptomatic or Positive Testing), Previous Stroke or TIA, Smoker, Peripheral Artery Disease; Aortic Aneurysm | No | No | Non death |
| 70 | 2612967H | Female | Hypertension, Hypothyroidism, Coronary Artery Disease (Symptomatic or Positive Testing), Heart Failure, Smoker, Cancer, Depression | No | No | Non death |
| 37 | 14142905G | Female | Hypertension, Diabetes Mellitus, Hypothyroidism, Chronic Kidney Disease, Cirrhosis; | No | No | Non death |
| 80 | 13951231C | Male | Hypertension, Diabetes Mellitus, Hypothyroidism, Coronary Artery Disease (Symptomatic or Positive Testing), Chronic Obstructive Pulmonary Disease (COPD, emphysema) | No | No | Non death |
| 70 | 13905114I | Female | Hypertension, ESTENOSE GASTRICA; FLUTTER ATRIAL; Chronic Hepatitis C; Epilepsy | No | No | Non death |
| 63 | 2429472C | Male | Hypertension, Diabetes Mellitus, Dyslipidemia, Chronic Obstructive Pulmonary Disease (COPD, emphysema), Benign Prostatic Hyperplasia | No | No | Non death |
| 72 | 13760344F | Male | Heart Failure, Chronic Obstructive Pulmonary Disease (COPD, emphysema), Dementia, Atrial Fibrillation; | No | No | Non death |
| 56 | 3221019J | Female | Hypertension, Diabetes Mellitus, Coronary Artery Disease (Symptomatic or Positive Testing), Heart Failure, Depression | No | No | Non death |
| 83 | 90936120K | Male | Chronic Kidney Disease, Benign Prostatic Hyperplasia | No | No | Non death |
| 55 | 14055287F | Female | Hypertension, Diabetes Mellitus, Dyslipidemia, Cancer, Epilepsy; Depression; Common Variable Immunodeficiency | No | No | Non death |
| 43 | 14016128B | Female | Hypertension, Diabetes Mellitus, Hypothyroidism, Asthma, Depression | No | No | Non death |
| 83 | 55400748E | Female | Hypertension, Dyslipidemia, Hypothyroidism, Chronic Kidney Disease, Chronic Obstructive Pulmonary Disease (COPD, emphysema), Cancer | No | Yes | Non death |
| 58 | 13914124E | Female | Hypertension, Diabetes Mellitus, Dyslipidemia, Coronary Artery Disease (Symptomatic or Positive Testing), Heart Failure | No | No | Non death |
| 22 | 14119178I | Female | Systemic Lupus | No | No | Non death |
| 64 | 13706024H | Male | Diabetes Mellitus, Hypothyroidism, Chronic Kidney Disease, Coronary Artery Disease (Symptomatic or Positive Testing), Heart Failure | No | No | Non death |
| 67 | 13570215D | Female | Hypertension, Hypothyroidism, Chronic Obstructive Pulmonary Disease (COPD, emphysema) | No | No | Non death |
| 40 | 13613347C | Female | Hypertension, Diabetes Mellitus, Dyslipidemia, Hypothyroidism, Asthma, Antiphospholipid Syndrome; | No | No | Non death |
| 69 | 13768124B | Male | Osteoporosis, Cancer, Deep Venous Thrombosis DE REPETIÇÃO; Depression | No | No | Non death |
| 70 | 13940033I | Female | Coronary Artery Disease (Symptomatic or Positive Testing), Heart Failure, Chronic Obstructive Pulmonary Disease (COPD, emphysema), Osteoporosis, Lung Interstitial Disease related to Smoke,Lichen Sclerosus, Crossed Renal Ectopy, Atrophic Pangastritis | No | No | Non death |
| 80 | 3302818A | Female | Hypertension, Diabetes Mellitus, Dyslipidemia, Hypothyroidism, Osteoporosis, Previous Stroke or TIA, Cataract, | No | No | Non death |
| 70 | 2594840G | Female | Hypertension, Dyslipidemia, Hypothyroidism, Osteoarthritis , Cancer, Osteopenia ,Deep Venous Thrombosis EM MMII, Lombalgia Ciática, Obesity Grau II, Pre Diabetes, Glaucoma | No | No | Non death |
| 77 | 5270555G | Female | Hypertension, Diabetes Mellitus, Dyslipidemia, Hypothyroidism, Heart Failure, Bilateral Cataract | No | No | Non death |
| 62 | 13534721K | Male | Hypertension, Diabetes Mellitus, Dyslipidemia, Chronic Kidney Disease, Class III Obesity | No | No | Non death |
| 86 | 2153610D | Female | Hypertension, Dyslipidemia, Heart Failure, Chagas Disease, Atrial Fibrillation, | No | No | Non death |
| 50 | 14102069A | Male | Hypertension, Diabetes Mellitus, Dyslipidemia, Chronic Kidney Disease, Heart Failure | No | No | Non death |
| 39 | 13741039A | Female | Systemic Lupus | No | No | Non death |
| 86 | 2257613G | Female | Hypertension, Previous Stroke or TIA, Diverticulosis, Pulmonary Embolism,Urinary Incontinence, Bilateral transtibial Amputation due to Leprosy | No | No | Non death |
| 75 | 13647530K | Female | Hypertension, Diabetes Mellitus, Dyslipidemia, Coronary Artery Disease (Symptomatic or Positive Testing), Heart Failure, Asthma, Obesity, Gastroesophageal Reflux, De1pression, Osteopenia, Severe Obstructive Sleep Apnea , Tension Type Headache, Constipation | No | No | Non death |
| 72 | 3319321F | Male | Hypertension, Diabetes Mellitus, Dyslipidemia, Chronic Kidney Disease, Coronary Artery Disease (Symptomatic or Positive Testing), Previous Stroke or TIA, Depression, Aortic Aneurysm | No | No | Non death |
| 89 | 2775356K | Female | Hypertension, Diabetes Mellitus, Dyslipidemia, Hypothyroidism | No | Yes | Non death |
| 87 | 5014229J | Female | Hypertension, Chronic Kidney Disease, Heart Failure, Osteoporosis, Chronic Pulmonary Embolism; Carotid Aneurysm | No | No | Non death |
| 66 | 14055017B | Male | Diabetes Mellitus, Dyslipidemia, Hypothyroidism, Peripheral Artery Obstruction | No | No | Non death |
| 51 | 13822494E | Female | Diabetes Mellitus | No | No | Non death |
| 55 | 13794155E | Female | Hypertension, Diabetes Mellitus, Dyslipidemia, Antisynthetase Syndrome; | No | No | Non death |
| 59 | 13683921D | Female | Hypertension, Hypothyroidism, Rheumatoid Arthritis | No | No | Non death |
| 69 | 14147330B | Male | Diabetes Mellitus, Smoker, Chronic Pancreatitis; | No | No | Non death |
| 64 | 90899135C | Male | Diabetes Mellitus, Cirrhosis | No | No | Non death |
| 73 | 14112794G | Female | Hypertension, Diabetes Mellitus, Dyslipidemia, Chronic Kidney Disease, Previous Stroke or TIA, Dementia,Normal Pressure Hydrocephalus | No | No | Non death |
| 82 | 55707717C | Male | Hypertension, Dyslipidemia, Chronic Kidney Disease, Osteoporosis, Abdominal Aortic Aneurysm | No | No | Non death |
| 65 | 88230772G | Male | Hypertension, Diabetes Mellitus, Chronic Kidney Disease, Heart Failure, Chronic Obstructive Pulmonary Disease (COPD, emphysema), Smoker | No | No | Death |
| 95 | 13463207C | Female | Hypertension, Dyslipidemia, Hypothyroidism, Chronic Kidney Disease, Heart Failure, Osteoporosis, Cancer | No | Yes | Death |
| 74 | 13698374A | Male | Hypertension, Hypothyroidism, Coronary Artery Disease (Symptomatic or Positive Testing), Heart Failure, Ulcerative Colitis; Cirrhosis | No | No | Death |
| 65 | 3198555K | Female | Hypertension, Diabetes Mellitus, Dyslipidemia, Coronary Artery Disease (Symptomatic or Positive Testing), Heart Failure, Chronic Obstructive Pulmonary Disease (COPD, emphysema), Osteoarthritis , Osteoporosis, Cancer | No | No | Death |
| 76 | 13926468E | Female | Hypertension, Diabetes Mellitus, Chronic Kidney Disease, Coronary Artery Disease (Symptomatic or Positive Testing), Heart Failure, Cancer | No | No | Death |
| 74 | 3292481K | Female | Hypertension, Diabetes Mellitus, Dyslipidemia, Hypothyroidism, Chronic Kidney Disease, Coronary Artery Disease (Symptomatic or Positive Testing), Osteoarthritis | No | Yes | Death |
| 68 | 3166877H | Male | Hypertension, Diabetes Mellitus, Dyslipidemia, Hypothyroidism, Chronic Kidney Disease, Coronary Artery Disease (Symptomatic or Positive Testing), Chronic Obstructive Pulmonary Disease (COPD, emphysema), Previous Stroke or TIA, Dementia, Peripheral Artery Obstruction | No | No | Death |
| 83 | 3130589H | Female | Hypertension, Diabetes Mellitus, Dyslipidemia, Chronic Kidney Disease, Coronary Artery Disease (Symptomatic or Positive Testing), Heart Failure, Osteoarthritis , Osteoporosis, Pulmonary Hypertension; Deep Venous Thrombosis; Hyperthyroidism | No | No | Death |
| 58 | 14169272C | Male | Hypertension, Chronic Kidney Disease, Coronary Artery Disease (Symptomatic or Positive Testing), Heart Failure, Anemia (with previous prescription of medical treatment or transfusion) | No | No | Death |
| 72 | 7038282F | Male | Hypertension, Diabetes Mellitus, Chronic Kidney Disease, Coronary Artery Disease (Symptomatic or Positive Testing), Heart Failure, Abdominal Aortic Aneurysm; Benign Prostatic Hyperplasia | No | No | Death |
| 80 | 2577785B | Female | Hypertension, Diabetes Mellitus | No | No | Death |
| 56 | 14111795K | Female | Hypertension, Diabetes Mellitus, Chronic Kidney Disease, Heart Failure, Pulmonary Hypertension | No | No | Death |
| 77 | 13740264E | Female | Asthma, Osteoporosis | No | No | Non death |
| 17 | 14142412E | Female | Diabetes Mellitus | No | No | Non death |
| 27 | 90724483H | Male | Inferior Vena Cava Agenesis; Deep Venous Thrombosis | No | No | Non death |
| 79 | 55385505F | Male | Dyslipidemia, Hypothyroidism, Coronary Artery Disease (Symptomatic or Positive Testing), Cancer, Anemia (with previous prescription of medical treatment or transfusion), Abdominal Aortic Aneurysm | No | No | Non death |
| 90 | 13982513E | Female | Hypertension, Diabetes Mellitus, Chronic Kidney Disease, Heart Failure, Osteoporosis, Anemia (with previous prescription of medical treatment or transfusion), Chronic Atrial Fibrillation | No | Yes | Non death |
| 84 | 5079392H | Male | Hypertension, Diabetes Mellitus, Chronic Kidney Disease, Coronary Artery Disease (Symptomatic or Positive Testing), Cancer | No | No | Non death |
| 83 | 13917246K | Female | Hypertension, Diabetes Mellitus, Dyslipidemia, Hypothyroidism, Chronic Kidney Disease, Dementia | No | No | Non death |
| 57 | 14076557H | Male | Hypertension, Diabetes Mellitus, Dyslipidemia, Previous Stroke or TIA | No | No | Non death |
| 82 | 90768634C | Female | Hypertension, Diabetes Mellitus, Coronary Artery Disease (Symptomatic or Positive Testing), Heart Failure, saccular ascending aortic aneurysm | No | No | Death |
| 79 | 14130342E | Male | Hypertension, Chronic Kidney Disease, Heart Failure, Chronic Atrial Fibrillation; Benign Prostatic Hyperplasia | No | No | Death |
| 80 | 90914860D | Female | Hypertension, Diabetes Mellitus, Hypothyroidism, Chronic Kidney Disease, Coronary Artery Disease (Symptomatic or Positive Testing), Heart Failure, Chronic Pulmonary Embolism | No | No | Death |
| 74 | 2855708B | Male | Hypertension, Dyslipidemia, Coronary Artery Disease (Symptomatic or Positive Testing), Heart Failure, Chronic Obstructive Pulmonary Disease (COPD, emphysema), Benign Prostatic Hyperplasia; Barrett Esophagus | No | No | Death |
| 80 | 55734950I | Female | Diabetes Mellitus, Dyslipidemia, Hypothyroidism, Chronic Kidney Disease, Coronary Artery Disease (Symptomatic or Positive Testing), Heart Failure, Previous Stroke or TIA, Chronic Atrial fibrillation | No | No | Death |
| 90 | 2425796A | Female | Hypertension, Heart Failure, Osteoporosis, Previous Stroke or TIA, Dementia | No | No | Death |
| 52 | 2166053C | Female | Hypertension, Diabetes Mellitus, Erosive Gastritis; Thrombocytopenia | No | No | Death |
| 89 | 2015001G | Female | Chronic Kidney Disease, Osteoporosis, Sheehan Syndrome | No | No | Death |
| 62 | 55766604D | Female | Hypertension, Diabetes Mellitus, Dyslipidemia, Chronic Kidney Disease, Heart Failure, Chronic Obstructive Pulmonary Disease (COPD, emphysema) | No | No | Death |
| 84 | 88221371C | Female | Hypertension, Chronic Kidney Disease, Osteoporosis, Dementia, Pulmonary Hypertension; Chronic Atrial fibrillation | No | No | Death |
| 79 | 13983613H | Female | Diabetes Mellitus | Yes | No | Death |
| 54 | 3285488F | Female | Hypertension, Diabetes Mellitus, Coronary Artery Disease (Symptomatic or Positive Testing), Previous Stroke or TIA, Cancer | Yes | No | Death |
| 47 | 3570452I | Female | Hypertension, Hypothyroidism, Asthma, Rheumatoid Arthritis, Depression | Yes | No | Death |
| 70 |  | Female | Hypertension, Dyslipidemia, Rheumatoid Arthritis | Yes | No | Death |
| 59 | 5020973I | Male | Hypertension, Diabetes Mellitus, Dyslipidemia, Chronic Kidney Disease, Gout | Yes | No | Death |
| 86 | 14133799G | Male | Chronic Kidney Disease, Heart Failure, Previous Stroke or TIA, Epilepsy | Yes | No | Death |
| 76 | 2661393B | Female | Rheumatoid Arthritis, HIV | Yes | No | Death |
| 72 | 5216831K | Male | Hypertension, Diabetes Mellitus, Dyslipidemia, Hypothyroidism, Chronic Kidney Disease | Yes | No | Death |
| 52 | 55558970G | Female | Hypothyroidism, Chronic Kidney Disease, Heart Failure, Atrial Fibrillation | Yes | No | Death |
| 79 | 14152497C | Female | Diabetes Mellitus, Hypothyroidism, Heart Failure, Atrial Fibrillation, Pulmonary Hypertension | Yes | Yes | Death |
| 70 | 3274053C | Female | Diabetes Mellitus, Dyslipidemia, Hypothyroidism, Heart Failure, Cancer | Yes | No | Death |
| 66 | 3036414D | Female | Hypertension, Diabetes Mellitus, Osteoarthritis | Yes | No | Death |
| 74 | 2602478H | Female | Hypertension, Diabetes Mellitus, Hypothyroidism, Coronary Artery Disease (Symptomatic or Positive Testing), Chronic Obstructive Pulmonary Disease (COPD, emphysema), Osteoarthritis , Previous Stroke or TIA | Yes | No | Death |
| 88 | 2183779J | Female | Hypertension, Hypothyroidism, Osteoporosis | Yes | No | Death |
| 57 | 14047825I | Female | Hypothyroidism, Fibromyalgia | Yes | No | Death |
| 68 | 14010201H | Female | Hypertension, Diabetes Mellitus, Chronic Kidney Disease, Coronary Artery Disease (Symptomatic or Positive Testing), Heart Failure, Previous Stroke or TIA | No | No | Non death |
| 45 | 13817862C | Female | Hypertension, Diabetes Mellitus, Dyslipidemia, Hypothyroidism | Yes | No | Non death |
| 25 | 14116501C | Female | Hypothyroidism | Yes | No | Non death |
| 76 | 7005463C | Female | Hypertension, Dyslipidemia, Hypothyroidism, Osteoarthritis | Yes | No | Non death |
| 35 | 13979389A | Male | Behçet Disease | Yes | No | Non death |
| 45 | 13965491F | Female | Hypertension, Graves Disease, Hypothyroidism due to Therapy with Iodine | Yes | No | Non death |
| 53 | 13982652A | Male | Hypertension, Diabetes Mellitus, Dyslipidemia, Coronary Artery Disease (Symptomatic or Positive Testing), Cancer | Yes | No | Non death |
| 59 | 13846662I | Male | Hypertension, Diabetes Mellitus, Dyslipidemia, Cancer | Yes | No | Non death |
| 79 | 13898368H | Female | Coronary Artery Disease (Symptomatic or Positive Testing), Polymyalgia Rheumatica , Psoriasis, Psoriatic Arthritis | Yes | No | Non death |
| 34 | 6029041C | Female | Diabetes Mellitus, Hyperthyroidism, Systemic Sclerosis | Yes | No | Non death |
| 50 | 13891817F | Female | Dyslipidemia, Hypothyroidism, Rheumatoid Arthritis, Fibromyalgia | Yes | No | Non death |
| 69 | 13913651F | Female | Hypertension, Diabetes Mellitus, Dyslipidemia | Yes | No | Non death |
| 69 | 13694144E | Male | Hypertension, Chronic Kidney Disease | Yes | No | Non death |
| 59 | 13867520J | Female | Rheumatoid Arthritis | Yes | No | Non death |
| 68 | 3057345C | Male | Hypertension, Diabetes Mellitus, Dyslipidemia, Chronic Kidney Disease | Yes | No | Non death |
| 62 | 3066108H | Female | Hypertension, Diabetes Mellitus, Dyslipidemia | Yes | No | Non death |
| 47 | 14128589J | Female | Hypertension, Graves Disease; Epilepsy | Yes | No | Non death |
| 45 | 2470552I | Female | Diabetes Mellitus, Hypothyroidism, Asthma | Yes | No | Non death |
| 35 | 13699883G | Female | Systemic Lupus | Yes | No | Non death |
| 17 | 14134533A | Female | Diabetes Mellitus | Yes | No | Non death |
| 74 | 55726276J | Female | Hypertension, Diabetes Mellitus, Dyslipidemia, Chronic Kidney Disease, Osteoarthritis , Previous Stroke or TIA | Yes | No | Non death |
| 73 | 5188737H | Male | Hypertension, Diabetes Mellitus, Dyslipidemia, Chronic Kidney Disease, Coronary Artery Disease (Symptomatic or Positive Testing), Heart Failure | Yes | No | Non death |
| 63 | 2530297J | Female | Hypertension, Osteoarthritis , Rheumatoid Arthritis, Anemia (with previous prescription of medical treatment or transfusion) | Yes | No | Non death |
| 27 | 13944232D | Male | Diabetes Mellitus | Yes | No | Non death |
| 63 | 2543358G | Female | Hypertension, Diabetes Mellitus, Dyslipidemia, Paget Disease | Yes | No | Non death |
| 61 | 2050683B | Female | Hypertension, Dyslipidemia, Rheumatoid Arthritis | Yes | No | Non death |
| 85 | 3355224C | Female | Hypertension, Dyslipidemia, Hypothyroidism, Chronic Kidney Disease, Coronary Artery Disease (Symptomatic or Positive Testing), Heart Failure, Osteoarthritis , Depression | Yes | No | Non death |
| 47 | 13784913D | Female | Hypothyroidism, Depression | Yes | No | Non death |
| 35 | 90586145I | Male | Anemia (with previous prescription of medical treatment or transfusion), Depression;Malnutrition | Yes | No | Non death |
| 52 | 90741086B | Male | Hypertension, Diabetes Mellitus, Heart Failure, Previous Stroke or TIA, Cancer, Paroxysmal Atrial Fibrillation ; Obstructive Sleep Apnea | Yes | No | Non death |
| 58 | 89011420E | Female | Dyslipidemia, Hypothyroidism, Depression | Yes | No | Non death |
| 36 | 14089759J | Female | Diabetes Mellitus, Systemic Lupus, Smoker | Yes | No | Non death |
| 60 | 13616749C | Female | Dyslipidemia, Hypothyroidism, Depression | Yes | No | Non death |
| 32 | 14065209K | Female | Systemic Lupus | Yes | No | Non death |
| 64 | 13994029K | Male | Diabetes Mellitus, Dyslipidemia | Yes | No | Non death |
| 72 | 3077428A | Female | Heart Failure, Hyperthyroidism; Atrial Fibrillation | Yes | No | Non death |
| 59 | 2893676F | Female | Hypertension, Diabetes Mellitus, Chronic Obstructive Pulmonary Disease (COPD, emphysema) | Yes | No | Non death |
| 51 | 13935237K | Female | Systemic Lupus, Depression | Yes | No | Non death |
| 60 | 13894840K | Female | Hypertension, Diabetes Mellitus, Dyslipidemia, Osteoarthritis , Depression | Yes | No | Non death |
| 75 | 14049036E | Female | Hypertension, Diabetes Mellitus, Chronic Obstructive Pulmonary Disease (COPD, emphysema), Osteoarthritis , Previous Stroke or TIA, Adrenal Insufficiency | Yes | No | Non death |
| 69 | 13519049A | Female | Hypertension, Diabetes Mellitus, Heart Failure | Yes | No | Non death |
| 64 | 13942838F | Female | Hypertension, Dyslipidemia, Asthma, Angioedema | Yes | No | Non death |
| 61 | 13778693K | Female | Osteoporosis, Depression; Autoimmune Disease | Yes | No | Non death |
| 66 | 2171508A | Female | Hypertension, Diabetes Mellitus, Dyslipidemia, Previous Stroke or TIA, Anemia (with previous prescription of medical treatment or transfusion), Chronic Hepatitis C; Central Nervous Demyelination | Yes | No | Non death |
| 68 | 2800844A | Female | Hypertension, Diabetes Mellitus, Dyslipidemia, Coronary Artery Disease (Symptomatic or Positive Testing), Heart Failure, Paripheral Arterial Obstruction | Yes | No | Non death |
| 51 | 14031808D | Female | Hypertension, Dyslipidemia, Heart Failure, Anemia (with previous prescription of medical treatment or transfusion), Depression | Yes | No | Non death |
| 71 | 13807588I | Male | Hypertension, Dyslipidemia, Chronic Kidney Disease, Chronic Obstructive Pulmonary Disease (COPD, emphysema) | Yes | No | Non death |
| 69 | 5247520D | Female | Hypertension, Diabetes Mellitus, Hypothyroidism, Heart Failure, Asthma | Yes | No | Non death |
| 54 | 3244499I | Female | Diabetes Mellitus, Osteoarthritis , Osteoporosis, Rheumatoid Arthritis | Yes | No | Non death |
| 46 | 77111329I | Male | Hypertension, Diabetes Mellitus, Chronic Kidney Disease, Cryptogenic Cirrhosis | Yes | No | Non death |
| 68 | 13986463G | Female | Hypertension, Dyslipidemia, Coronary Artery Disease (Symptomatic or Positive Testing), Heart Failure, Smoker | Yes | No | Non death |
| 41 | 5237549F | Male | Ulcerative Colitis | Yes | No | Non death |
| 51 | 44127801B | Female | Previous Stroke or TIA, PROVAVEL Antiphospholipid Syndrome | Yes | No | Non death |
| 65 | 5250725I | Female | Hypertension, Diabetes Mellitus, Chronic Kidney Disease, Coronary Artery Disease (Symptomatic or Positive Testing) | Yes | No | Non death |
| 43 | 55391758E | Male | Hypertension, Diabetes Mellitus, Dyslipidemia | Yes | No | Non death |
| 64 | 3214810K | Female | Hypertension, Osteoarthritis , Osteoporosis, Chronic Venous Disease | Yes | No | Non death |
| 31 | 13743997E | Male | Asthma, Osteoporosis, Rheumatoid Arthritis, Epilepsy; Autism Spectrum | Yes | No | Non death |
| 54 | 14082420I | Female | Hypertension, Diabetes Mellitus, Dyslipidemia, Smoker | Yes | No | Non death |
| 53 | 14092512K | Female | Hypertension, Diabetes Mellitus, Dyslipidemia, Hypothyroidism | Yes | No | Non death |
| 62 | 13771466E | Female | Hypertension, Dyslipidemia, Rheumatoid Arthritis | Yes | No | Non death |
| 41 | 13984418E | Male | Hypertension, Diabetes Mellitus, Dyslipidemia, Asthma | Yes | No | Non death |
| 59 | 13895775G | Female | Hypertension, Diabetes Mellitus, Dyslipidemia, Previous Stroke or TIA | Yes | No | Non death |
| 70 | 3331586C | Female | Hypertension, Diabetes Mellitus, Dyslipidemia, Coronary Artery Disease (Symptomatic or Positive Testing), Osteoarthritis , Osteoporosis, Depression | Yes | No | Non death |
| 62 | 13689019E | Female | Dyslipidemia, Osteoarthritis , Osteoporosis, Rheumatoid Arthritis, Peripheral Arterial Obstruction | Yes | No | Non death |
| 59 | 2131547c | Female | Hypertension, Hypothyroidism, Obstructive Sleep Apnea | Yes | No | Non death |
| 61 | 55715514g | Male | Hypertension, Diabetes Mellitus, Previous Stroke or TIA, Mechanic Aortic Valve Replacement | Yes | No | Non death |
| 57 | 13807990i | Female | Diabetes Mellitus, Dyslipidemia | Yes | No | Non death |
| 27 | 13971606a | Female | Systemic Lupus, Smoker | Yes | No | Non death |
| 65 | 3155110F | Male | Hypertension, Diabetes Mellitus, Dyslipidemia, Heart Failure, Anemia (with previous prescription of medical treatment or transfusion), Peripheral Arterial Disease | Yes | No | Non death |
| 56 | 13884540G | Female | Hypertension, Dyslipidemia, Sarcoidosis, Depression | Yes | No | Non death |
| 70 | 3230930I | Female | Hypertension, Diabetes Mellitus, Hypothyroidism | Yes | No | Non death |
| 35 | 13608086G | Female | Dyslipidemia, Rheumatoid Arthritis, Epilepsy | Yes | No | Non death |
| 53 | 13944611F | Female | Hypertension, Diabetes Mellitus, Dyslipidemia, Osteoarthritis | Yes | No | Non death |
| 54 | 14096814J | Female | Dyslipidemia, Histiocytosis X | Yes | No | Non death |
| 71 | 3318558I | Male | Hypertension, Diabetes Mellitus, Coronary Artery Disease (Symptomatic or Positive Testing), Previous Stroke or TIA, Atrial Fibrillation | Yes | No | Non death |
| 69 | 14054539A | Male | Hypertension, Diabetes Mellitus, Dyslipidemia, Hypothyroidism, Chronic Kidney Disease, Coronary Artery Disease (Symptomatic or Positive Testing), ENCEFALOPATIA DE WERNICK | Yes | No | Non death |
| 70 | 13688054C | Female | Hypertension, Diabetes Mellitus, Hypothyroidism | Yes | No | Non death |
| 62 | 13777070E | Female | Hypertension, Diabetes Mellitus, Dyslipidemia, Hypothyroidism, Chronic Kidney Disease, Peripheral Arterial Disease | Yes | No | Non death |
| 62 | 14021668K | Male | Hypertension, Dyslipidemia, Henoch-Schonlein Purpura | Yes | No | Non death |
| 59 | 13915645F | Female | Diabetes Mellitus, Heart Failure, Obstructive Sleep Apnea | Yes | No | Non death |
| 55 | 2176288H | Female | Hypertension, Diabetes Mellitus, Dyslipidemia, Previous Stroke or TIA, Psoriasic Arthritis | Yes | No | Non death |
| 42 | 14168742H | Female | Smoker, Hyperthyroidism | Yes | No | Non death |
| 59 | 14002541B | Male | Hypertension, Dyslipidemia, Rheumatoid Arthritis | Yes | No | Non death |
| 59 | 14118140I | Female | Hypertension, Diabetes Mellitus, Rheumatoid Arthritis, Smoker | Yes | No | Non death |
| 48 | 14109077I | Female | Hypertension, Heart Failure, Hyperthyroidism; Schistosomiasis Related Portal Hypertension | Yes | No | Non death |
| 52 | 13743805E | Female | Hypertension | Yes | No | Non death |
| 60 | 14012606J | Male | Diabetes Mellitus, Hypothyroidism, Chronic Kidney Disease, Osteoporosis, Chronic Pancreatitis | Yes | No | Non death |
| 47 | 14159925D | Female | Hypertension, Deep Venous Thrombosis | Yes | No | Non death |
| 72 | 13631645B | Female | Hypertension, Diabetes Mellitus, Hypothyroidism, Chronic Obstructive Pulmonary Disease (COPD, emphysema), Osteoporosis | Yes | No | Non death |
| 48 | 13934115F | Female | Systemic Lupus, Smoker, Depression | Yes | No | Non death |
| 56 | 33409012H | Male | Hypertension, Rheumatoid Arthritis, Sjogren Disease | Yes | No | Non death |
| 31 | 14025002F | Female | Systemic Lupus | Yes | No | Non death |
| 83 | 3160105A | Female | Hypertension, Diabetes Mellitus, Dyslipidemia, Hypothyroidism, Osteoporosis, Smoker | Yes | No | Non death |
| 67 | 13470010B | Female | Hypertension, Diabetes Mellitus, Dyslipidemia, Heart Failure, Rheumatoid Arthritis | Yes | No | Non death |
| 62 | 13997507F | Male | Hypertension, Diabetes Mellitus, Dyslipidemia, Hypothyroidism, Previous Stroke or TIA | Yes | No | Non death |
| 64 | 13806363K | Female | Hypertension, Diabetes Mellitus, Dyslipidemia, Previous Stroke or TIA | Yes | No | Non death |
| 63 | 2216369E | Female | Diabetes Mellitus, Dyslipidemia, Hypothyroidism, Asthma | Yes | No | Non death |
| 73 | 2338946E | Female | Hypertension, Diabetes Mellitus, Dyslipidemia, Hypothyroidism, Chronic Kidney Disease, Heart Failure, Osteoarthritis , Paroxysmal Atrial Fibrillation | Yes | No | Non death |
| 60 | 14061275C | Male | Diabetes Mellitus, Skin Secondary Amyloidosis | Yes | No | Non death |
| 35 | 13745374F | Male | Systemic Lupus | Yes | No | Non death |
| 84 | 13964329F | Female | Hypertension, Diabetes Mellitus, Dyslipidemia, Hypothyroidism, Celiac Artery Trunk Stenosis | Yes | Yes | Non death |
| 66 | 13997651D | Female | Hypertension, Diabetes Mellitus, Dyslipidemia, Anemia (with previous prescription of medical treatment or transfusion) | Yes | No | Non death |
| 62 | 13992718H | Female | Hypertension, Diabetes Mellitus, Dyslipidemia, Hypothyroidism, Obstructive Sleep Apnea | Yes | No | Non death |
| 63 | 13932829C | Male | Hypertension, Dyslipidemia, Chronic Kidney Disease, Coronary Artery Disease (Symptomatic or Positive Testing), Heart Failure | Yes | No | Non death |
| 71 | 2974743C | Female | Hypertension, Osteoarthritis , Rheumatoid Arthritis | Yes | No | Non death |
| 61 | 13441010D | Male | Diabetes Mellitus, Chronic Kidney Disease, Coronary Artery Disease (Symptomatic or Positive Testing) | Yes | No | Non death |
| 43 | 13592476J | Female | Systemic Lupus | Yes | No | Non death |
| 68 | 55461645D | Female | Coronary Artery Disease (Symptomatic or Positive Testing), Anemia (with previous prescription of medical treatment or transfusion) | Yes | No | Non death |
| 65 | 55554113H | Female | Hypertension, Dyslipidemia, Coronary Artery Disease (Symptomatic or Positive Testing), Heart Failure | Yes | No | Non death |
| 70 | 2291444K | Female | Hypertension, Dyslipidemia, Osteoporosis, Systemic Lupus, Previous Stroke or TIA | Yes | No | Non death |
| 63 | 13679848D | Female | Hypertension, Diabetes Mellitus, Dyslipidemia | Yes | No | Non death |
| 68 | 14078123E | Male | Hypertension, Dyslipidemia, Hypothyroidism, Heart Failure, Paroxysmal Atrial Fibrillation ; | Yes | No | Non death |
| 69 | 13445536G | Female | Hypothyroidism, Asthma, Osteoarthritis , NASH Cirrhosis, Bariatric Surgery | Yes | No | Non death |
| 68 | 13685623D | Female | Hypertension, Diabetes Mellitus, Dyslipidemia, Osteoarthritis , Obesity, Osteopenia, Dermatomyositis | Yes | No | Non death |
| 37 | 14171191C | Female | Dyslipidemia, Osteoporosis, Systemic Lupus, Depression | Yes, No | No | Non death |
| 68 | 3244525H | Male | Hypertension, Dyslipidemia, Chronic Obstructive Pulmonary Disease (COPD, emphysema), Vocal Folds Surgery, Alcohol Use Disorder diario, Previous Smoker | Yes | No | Non death |
| 65 | 13930020J | Male | Hypertension, Diabetes Mellitus, Hypothyroidism, Chronic Kidney Disease, Heart Failure, Chronic Ulcers in Lower Members, Secondary Hyperparathyroidism, Obstructive Sleep Apnea , Cataract, Allergic Rhinitis. | Yes | No | Non death |
| 47 | 13755379F | Male | Diabetes Mellitus, Carpal Tunnel Syndrome, PTI cronica, Gastroesophageal Reflux | Yes | No | Non death |
| 62 | 44125546G | Female | Hypertension, Diabetes Mellitus, Hypothyroidism, Osteoarthritis , Rheumatoid Arthritis, Osteopenia, Systemic Sclerosis, Sjögren Syndrome_ Raynaud phenomenon | Yes | No | Non death |
| 59 | 13931935E | Female | Hypertension, Diabetes Mellitus, Dyslipidemia, Hypothyroidism, Obesity, esteatose, hepática, Functional Dyspepsia., | Yes | No | Non death |
| 80 | 3325341C | Female | Hypertension, Diabetes Mellitus, Dyslipidemia, Coronary Artery Disease (Symptomatic or Positive Testing), Osteoarthritis , Previous Stroke or TIA, Fibromyalgia , Gastroesophageal Reflux | Yes | No | Non death |
| 79 | 3120127F | Male | Hypertension, Dyslipidemia, Hypothyroidism, Chronic Kidney Disease, Heart Failure, Osteoarthritis , Previous Stroke or TIA, Resistência insulínica, Gout, Tubular Adenomas with Low Grade Dysplasia | Yes | No | Non death |
| 63 | 13623524I | Male | Hypertension, Dyslipidemia, Ulcerative Colitis, Atrial Fibrillation, Osteopenia | Yes | No | Non death |
| 80 | 3279302D | Female | Diabetes Mellitus, Hypothyroidism, Osteoarthritis , Anemia Perniciosa, Fenômeno de Raynauld/Livedo reticular, | Yes | No | Non death |
| 41 | 13857349I | Female | Systemic Lupus, Depression, Herpes zoster | Yes | No | Non death |
| 65 | 13566415E | Female | Diabetes Mellitus, Dyslipidemia, Asthma, Gastroesophageal Reflux, Chronic Venous Insufficiency periférica. | Yes | No | Non death |
| 35 | 13610871F | Female | Hypertension, Systemic Sclerosis, Pulmonary Embolism | Yes | No | Non death |
| 36 | 14046939C | Female | Rheumatoid Arthritis, Depression | Yes | No | Non death |
| 62 | 3121668A | Male | Hypertension, Diabetes Mellitus, Dyslipidemia, Chronic Kidney Disease, Osteoarthritis , Xeroderma Pigmentosum with previous Basal Cell Carcinoma and Squamous Cell Carcinomacom | Yes | No | Non death |
| 63 | 2760254D | Female | Hypertension, Diabetes Mellitus, Dyslipidemia, Osteoarthritis , Depression, Obesity, Epilepsy | Yes | No | Non death |
| 37 | 6020149C | Male | Diabetes Mellitus, Dyslipidemia | Yes | No | Non death |
| 43 | 14125702D | Male | Dyslipidemia, Rheumatoid Arthritis, Prediabetes, Hepatic Steatosis | Yes | No | Non death |
| 59 | 13590683A | Female | Hypertension, Diabetes Mellitus, Dyslipidemia, Osteoarthritis , Gastroesophageal Reflux/Dyspepsia , Hypothyroidism , Chronic Venous Insufficiency , Fibromyalgia , Depression | Yes | No | Non death |
| 55 | 55758284C | Male | Hypothyroidism, Heart Failure, Chronic Obstructive Pulmonary Disease (COPD, emphysema), Osteoporosis, Systemic Sclerosis , Nonspecific Interstitial Pneumonia | Yes | No | Non death |
| 49 | 14083135H | Female | Hypertension, Previous Stroke or TIA, Fator V de Leiden, Anxiety Disorder, Leiomyomas, Obstructive Sleep Apnea , Obesity | Yes | No | Non death |
| 66 | 2138936K | Female | Hypertension, Diabetes Mellitus, Dyslipidemia, Coronary Artery Disease (Symptomatic or Positive Testing), Heart Failure, Chronic Obstructive Pulmonary Disease (COPD, emphysema), Osteoarthritis , Rheumatoid Arthritis, Dyspepsia, Glaucoma, Chronic Constipation, Depression. | Yes | No | Non death |
| 37 | 13862669J | Female | Systemic Lupus, Obesity, Migraine, Eating Disorder | Yes | No | Non death |
| 61 | 13998996C | Female | Hypertension, Rheumatoid Arthritis, Mechanical Polyarthralgia;Fibromyalgia , Hydroxychloroquine-Induced Maculopathy/Retinopathy | Yes | No | Non death |
| 75 | 14042765D | Male | Hypertension, Dyslipidemia, Chronic Obstructive Pulmonary Disease (COPD, emphysema), Benign Prostatic Hyperplasia, Insuficiencia venosa de MMII, Mild Obstructive Lung Disease. | Yes | No | Non death |
| 41 | 14109397B | Male | Hypertension, Diabetes Mellitus, Dyslipidemia, Chronic Kidney Disease | Yes | No | Non death |
| 63 | 3084970E | Male | Hypertension, Diabetes Mellitus, Dyslipidemia, Chronic Kidney Disease, Coronary Artery Disease (Symptomatic or Positive Testing), Heart Failure, Glaucoma e Cataract, Chronic Pancreatitis | Yes | No | Non death |
| 39 | 14100706D | Male | Diabetes Mellitus, Dyslipidemia | Yes | No | Non death |
| 77 | 90746916F | Female | Hypertension, Dyslipidemia, Hypothyroidism, Osteoporosis, Smoker | Yes, No | No | Non death |
| 57 | 7041491C | Male | Hypertension, Diabetes Mellitus, Dyslipidemia | Yes | No | Non death |
| 61 | 14055945C | Female | Hypertension, Diabetes Mellitus, Depression | Yes | No | Non death |
| 62 | 14093228I | Female | Hypertension, Diabetes Mellitus, Hypothyroidism, Obesity | Yes | No | Non death |
| 43 | 88239126H | Female | Diabetes Mellitus, Hypothyroidism, Chronic Obstructive Pulmonary Disease (COPD, emphysema) | Yes | No | Non death |
| 78 | 3233616F | Female | Hypertension, Dyslipidemia, Osteoporosis, Fibromyalgia | Yes | No | Non death |
| 64 | 13736987J | Female | Hypertension, Doença mista do tecido conjuntivo, Depression. | Yes | No | Non death |
| 80 | 2503152A | Male | Hypertension, Diabetes Mellitus, Dyslipidemia, Hypothyroidism | Yes | No | Non death |
| 63 | 14163553D | Female | Hypertension, Diabetes Mellitus, Dyslipidemia, Chronic Kidney Disease, Smoker | Yes | No | Non death |
| 48 | 14071329D | Female | Chronic Kidney Disease, Anemia (with previous prescription of medical treatment or transfusion) | Yes | No | Non death |
| 64 | 14011282K | Female | Hypertension, Diabetes Mellitus, Dyslipidemia, Hypothyroidism, Obesity | Yes | No | Non death |
| 58 | 13770338J | Female | Hypertension, Diabetes Mellitus, Dyslipidemia, Asthma | Yes | No | Non death |
| 69 | 13542288J | Female | Hypertension, Diabetes Mellitus, Osteoarthritis , Rheumatoid Arthritis | Yes | No | Non death |
| 27 | 14048643H | Male | Hypothyroidism, Systemic Lupus | Yes | No | Non death |
| 32 | 13968367I | Female | Diabetes Mellitus | Yes | No | Non death |
| 58 | 14112621B | Female | Hyperthyroidism, Depression | Yes | No | Non death |
| 66 | 14125199E | Female | Hypertension, Diabetes Mellitus, Dyslipidemia, Chronic Kidney Disease, Heart Failure, Chronic Obstructive Pulmonary Disease (COPD, emphysema) | Yes | No | Non death |
| 64 | 2798116D | Female | Hypertension, Dyslipidemia, Rheumatoid Arthritis, Previous Stroke or TIA | Yes | No | Non death |
| 70 | 3326708I | Female | Hypertension, Diabetes Mellitus, Hypothyroidism, Chronic Kidney Disease, Heart Failure | Yes | No | Non death |
| 27 | 13960684A | Male | Hypertension, Diabetes Mellitus, Dyslipidemia, Hypothyroidism | Yes | No | Non death |
| 56 | 3213329B | Female | Hypertension, Diabetes Mellitus, Dyslipidemia, Cancer | Yes | No | Non death |
| 46 | 3188096B | Female | Diabetes Mellitus, Chronic Hepatitis C, Depression | Yes | No | Non death |
| 49 | 2745049H | Male | Dyslipidemia, Heart Failure, Epilepsy | Yes | No | Non death |
| 35 | 14123822D | Male | Asthma, Systemic Lupus, Primary Adrenal Insufficiency | Yes | No | Non death |
| 53 | 13747356K | Female | Diabetes Mellitus, Heart Failure, Osteoarthritis , Rheumatoid Arthritis, Depression | Yes | No | Non death |
| 65 | 14083049E | Male | Hypertension, Diabetes Mellitus, Coronary Artery Disease (Symptomatic or Positive Testing), Previous Stroke or TIA | Yes | No | Non death |
| 82 | 33600857A | Male | Hypertension, Diabetes Mellitus, Previous Stroke or TIA, Cancer, Obesity | Yes | No | Non death |
| 73 | 14096618C | Male | Diabetes Mellitus, Coronary Artery Disease (Symptomatic or Positive Testing), Chronic Obstructive Pulmonary Disease (COPD, emphysema) | Yes | No | Non death |
| 18 | 14168972B | Female | Hypothyroidism, Systemic Lupus | Yes | No | Non death |
| 71 | 14132262D | Male | Diabetes Mellitus, Chronic Pancreatitis | Yes | No | Non death |
| 53 | 55756277B | Male | Hypertension, Diabetes Mellitus, Dyslipidemia, Chronic Kidney Disease, Coronary Artery Disease (Symptomatic or Positive Testing), Obesity | Yes | No | Non death |
| 38 | 88244423G | Female | Hypothyroidism, Rheumatoid Arthritis, Obesity | Yes | No | Non death |
| 73 | 2901778B | Female | Hypertension, Diabetes Mellitus, Dyslipidemia | Yes | No | Non death |
| 65 | 14080242H | Female | Hypertension, Dyslipidemia, Hypothyroidism | Yes | No | Non death |
| 61 | 13524639J | Female | Dyslipidemia, Rheumatoid Arthritis | Yes | No | Non death |
| 57 | 2712552C | Female | Hypertension, Diabetes Mellitus, Dyslipidemia, Osteoporosis | Yes | No | Non death |
| 63 | 13619400J | Male | Hypertension, Diabetes Mellitus, Dyslipidemia, Chronic Kidney Disease, Previous Stroke or TIA | Yes | No | Non death |
| 49 | 2401424B | Female | Hypertension, Dyslipidemia, Osteoarthritis , Depression | Yes | No | Non death |
| 58 | 13818957C | Male | Diabetes Mellitus, Smoker | Yes | No | Non death |
| 70 | 4003692J | Female | Hypertension, Diabetes Mellitus, Dyslipidemia, Chronic Kidney Disease, Heart Failure | Yes | No | Non death |
| 68 | 13511999A | Female | Hypertension, Diabetes Mellitus, Dyslipidemia, Hypothyroidism, Coronary Artery Disease (Symptomatic or Positive Testing), Heart Failure, Smoker | Yes | No | Non death |
| 58 | 3195016B | Female | Hypertension, Diabetes Mellitus | Yes | No | Non death |
| 66 | 2082323D | Female | Hypothyroidism, Osteoporosis, Systemic Sclerosis | Yes | No | Non death |
| 56 | 13738546G | Male | Hypertension, Chronic Obstructive Pulmonary Disease (COPD, emphysema), Addison Disease | Yes | No | Non death |
| 67 | 14064161J | Male | Chronic Kidney Disease, Chronic Obstructive Pulmonary Disease (COPD, emphysema) | Yes | No | Non death |
| 58 | 14103900I | Male | Hypertension, Diabetes Mellitus, Chronic Kidney Disease, Coronary Artery Disease (Symptomatic or Positive Testing), Heart Failure | Yes | No | Non death |
| 55 | 13977515F | Female | Hypertension, Diabetes Mellitus, Hypothyroidism | Yes | No | Non death |
| 71 | 90993425F | Male | Chronic Obstructive Pulmonary Disease (COPD, emphysema), Cancer, Anemia (with previous prescription of medical treatment or transfusion), Cirrhosis | Yes | No | Non death |
| 34 | 13743619F | Female | Hypertension, Previous Stroke or TIA, Systemic Lupus | Yes | No | Non death |
| 55 | 3363016E | Female | Dyslipidemia, Hypothyroidism, Rheumatoid Arthritis | Yes | No | Non death |
| 67 | 3379983C | Female | Hypertension, Diabetes Mellitus, Chronic Obstructive Pulmonary Disease (COPD, emphysema) | Yes | No | Non death |
| 58 | 2280704K | Female | Hypertension, Diabetes Mellitus, Dyslipidemia, Hypothyroidism, Osteoarthritis | Yes | No | Non death |
| 54 | 3276955J | Female | Heart Failure, Osteoporosis, Systemic Lupus | Yes | No | Non death |
| 34 | 14148431C | Male | Hypertension, Dyslipidemia, Chronic Kidney Disease, Heart Failure | Yes | No | Non death |
| 54 | 55448700E | Female | Osteoarthritis , Rheumatoid Arthritis, Smoker, Fibromyalgia , Depression, Anxiety Disorder | Yes | No | Non death |
| 58 | 88239941H | Female | Hypothyroidism, Chronic Kidney Disease, Coronary Artery Disease (Symptomatic or Positive Testing), Systemic Lupus, Previous Stroke or TIA, Smoker | Yes | No | Non death |
| 58 | 13813157E | Female | Hypertension, Diabetes Mellitus, Dyslipidemia, Heart Failure, Gastroesophageal Reflux | Yes | No | Non death |
| 52 | 14143767A | Male | Hypertension, Previous Stroke or TIA, Policitemia vera | Yes | No | Non death |
| 58 | 3158441B | Female | Hypertension, Diabetes Mellitus, Dyslipidemia, Osteoporosis, Depression | Yes | No | Non death |
| 66 | 13985649D | Male | Hypertension, Diabetes Mellitus, Dyslipidemia, Heart Failure, Chronic Obstructive Pulmonary Disease (COPD, emphysema), Osteoarthritis | Yes | No | Non death |
| 30 | 90826766D | Female | Rheumatoid Arthritis | Yes | No | Non death |
| 77 | 14063423B | Female | Hypertension, Diabetes Mellitus, Dyslipidemia, Previous Stroke or TIA | Yes | No | Non death |
| 78 | 13467288B | Female | Hypertension, Hypothyroidism, Coronary Artery Disease (Symptomatic or Positive Testing), Osteoporosis, Depression | Yes | No | Non death |
| 68 | 5283422B | Male | Hypertension, Diabetes Mellitus, Chronic Kidney Disease, Coronary Artery Disease (Symptomatic or Positive Testing), Chronic Obstructive Pulmonary Disease (COPD, emphysema), Sleep Apnea | Yes | No | Non death |
| 84 | 55529657I | Female | Hypertension, Dyslipidemia, Hypothyroidism, Chronic Kidney Disease, Coronary Artery Disease (Symptomatic or Positive Testing), Heart Failure | Yes | No | Non death |
| 69 | 13992955B | Male | Hypothyroidism, Coronary Artery Disease (Symptomatic or Positive Testing), Heart Failure, Chronic Obstructive Pulmonary Disease (COPD, emphysema) | Yes | No | Non death |
| 63 | 3294454G | Female | Hypertension, Hypothyroidism, Heart Failure, Rheumatoid Arthritis, Depression | Yes | No | Non death |
| 43 | 77131317B | Female | Anemia (with previous prescription of medical treatment or transfusion), Migraine, Obsessive-compulsive Disorder | Yes | No | Non death |
| 65 | 55556097G | Female | Hypertension, Dyslipidemia, Hipopituitarismo | Yes | No | Non death |
| 43 | 13932090A | Female | Diabetes Mellitus, Dyslipidemia, Systemic Lupus, Depression | Yes | No | Non death |
| 73 | 14027905A | Male | Infflamatory Bowel Syndrome, Gastroesophageal Reflux | Yes | No | Non death |
| 34 | 13912985H | Female | Systemic Lupus, Anxiety Disorder, Migraine | Yes | No | Non death |
| 64 | 55726086D | Male | Hypertension, Diabetes Mellitus, Dyslipidemia, Cancer, Epilpesia | Yes | No | Non death |
| 75 | 13495435D | Female | Hypertension, Diabetes Mellitus, Dyslipidemia, Hypothyroidism, Depression | Yes | No | Non death |
| 57 | 14156433E | Female | Hypertension, Diabetes Mellitus, Dyslipidemia | Yes | No | Non death |
| 62 | 13688865k | Male | Hypothyroidism, Smoker, Cancer | Yes | No | Non death |
| 41 | 4033367H | Female | Dyslipidemia, Chronic Obstructive Pulmonary Disease (COPD, emphysema), Osteoporosis | Yes | No | Non death |
| 40 | 14074372C | Female | Hypothyroidism, Systemic Lupus, Obesity | Yes | No | Non death |
| 57 | 13744243C | Male | Hypertension, Diabetes Mellitus | Yes | No | Non death |
| 62 | 14162518E | Male | Hypertension, Chronic Kidney Disease, Coronary Artery Disease (Symptomatic or Positive Testing) | Yes | No | Non death |
| 16 | 14096645H | Female | Diabetes Mellitus, Dyslipidemia | Yes | No | Non death |
| 21 | 6114746E | Female | Systemic Lupus | Yes | No | Non death |
| 62 | 13970559C | Male | Hypertension, Diabetes Mellitus, Dyslipidemia, Previous Stroke or TIA, Depression | Yes | No | Non death |
| 53 | 14003686I | Male | Chronic Obstructive Pulmonary Disease (COPD, emphysema), Previous Smoker, Cataract, Previous Substance Abuse | Yes | No | Non death |
| 68 | 90829765A | Female | Hypertension, Dyslipidemia, Hypothyroidism, Chronic Kidney Disease, Heart Failure, Previous Stroke or TIA, Smoker, Previous Smoker, Pre Diabetes | Yes | No | Non death |
| 67 | 13456353A | Female | Hypertension, Diabetes Mellitus, Dyslipidemia, Hypothyroidism, Asthma, Systemic Lupus, Esophageal Candidiasis | Yes | No | Non death |
| 74 | 3085400H | Female | Osteoarthritis , Systemic Lupus, Dizziness and Peripheral Vertigo | Yes | No | Non death |
| 63 | 2141095D | Female | Hypertension, Diabetes Mellitus, Dyslipidemia, Osteoarthritis , Dyspepsia // Gastroesophageal Reflux , Depression | Yes | No | Non death |
| 55 | 14112762G | Female | Hypertension, Diabetes Mellitus, Dyslipidemia, Hypothyroidism, Rheumatoid Arthritis | Yes | No | Non death |
| 85 | 13641947D | Male | Hypertension, Dyslipidemia, Chronic Kidney Disease, Asthma, Anemia,Chronic Venous Insufficiency, Peripferal Artery Disease/Obstruction, Cataract, Hypothyroidism, Chronic Obstructive Pulmonary Disease (COPD, Emphysema) | Yes | No | Non death |
| 60 | 13839161J | Male | Hypertension, Diabetes Mellitus, Dyslipidemia, Chronic Kidney Disease, Previous Smoker, anemia, Low Visual Acuity | Yes | No | Non death |
| 67 | 3342597J | Female | Hypertension, Diabetes Mellitus, Hypothyroidism, Chronic Kidney Disease, Osteoarthritis , Cancer, Multiple Myeloma , Previous Smoker, Breast Cancer | Yes | Yes | Non death |
| 53 | 14176140E | Female | Rheumatoid Arthritis, Cancer, Thyrotoxicosis , Thyroid Cancer | Yes | No | Non death |
| 58 | 14018030A | Male | Hypertension, Dyslipidemia, Chronic Kidney Disease, Previous Stroke or TIA, Smoker, Schizophrenia, Hipertiroidismo,Lithium induced Hyperparathyroidism | Yes | No | Non death |
| 52 | 14140078G | Female | Hypertension, Asthma, Rhinitis, Previous Smoker, urticaria crônica, ulcera de pressão. | Yes | No | Non death |
| 61 | 13950096D | Female | Hypertension, Asthma, Osteoarthritis , Depression, Systemic Lupus, Blastic Lesion in Tibia, Hepatic Steatosis, Glaucoma | Yes | No | Non death |
| 60 | 13876261H | Female | Dyslipidemia, Hypothyroidism, Fibromyalgia , Pseudoxanthoma Elasticum, Hyperthyroidism, Lung Obstructive Disease, Allergic Rhinitis | Yes | No | Non death |
| 77 | 14101315B | Male | Hypertension, Dyslipidemia, Pre Diabetes, Substance Abuse | Yes | No | Non death |
| 66 | 14147167E | Male | Diabetes Mellitus, Dyslipidemia, Hypothyroidism, Previous Smoker, Previous Alcohol Use Disorder | Yes | No | Non death |
| 60 | 3198378J | Male | Hypertension, Systemic Lupus, Previous Smoker | Yes | No | Non death |
| 81 | 2877136H | Female | Hypertension, Dyslipidemia, Chronic Obstructive Pulmonary Disease (COPD, emphysema), Osteopenia, Tireotoxicose, Glaucoma, | Yes | No | Non death |
| 68 | 55700384C | Male | Hypertension, Dyslipidemia, Chronic Kidney Disease, Heart Failure, Osteoarthritis , Previous Stroke or TIA, Obstructive Sleep Apnea , Diverticulosis, Allergic Rhinitis. | Yes | No | Non death |
| 61 | 2780748D | Female | Hypertension, Diabetes Mellitus, Previous Stroke or TIA | Yes | No | Non death |
| 80 | 13563512H | Male | Hypertension, Dyslipidemia, Chronic Kidney Disease, Heart Failure, Retinopatia hipertensiva grave , Gout, Osteopenia, Diverticulosis | Yes | No | Non death |
| 59 | 2134479C | Female | Hypertension, Dyslipidemia, Rheumatoid Arthritis, Permanent Pacemaker due to Cardiac Arrhytmia, Obesity, Pulmonary Embolism | Yes | No | Non death |
| 57 | 14040786G | Female | Heart Failure, Rheumatoid Arthritis, FA/ flutter, hipertireiodismo, | Yes | No | Non death |
| 47 | 90916064K | Female | Hypertension, Diabetes Mellitus, Heart Failure, Smoker, Substance Abuse | Yes | No | Non death |
| 65 | 2497726C | Female | Hypertension, Diabetes Mellitus, Dyslipidemia, Hyperthyroidism, Deep Venous Thrombosis e Fibromyalgia . | Yes | No | Non death |
| 77 | 2529185B | Female | Diabetes Mellitus, Dyslipidemia, Osteoarthritis ,Peripheral ARtery Disease/Obstruction , Cataract, Depression, Fibromyalgia , Osteopenia, Low Grade Tubular Adenomas | Yes | No | Non death |
| 65 | 14070045H | Female | Hypertension, Diabetes Mellitus, Hypothyroidism, Chronic Kidney Disease, Heart Failure, Anemia (with previous prescription of medical treatment or transfusion), Breast Lumps birads 4, Cataract, Osteopenia, | Yes | No | Non death |
| 61 | 2821956H | Female | Hypertension, Diabetes Mellitus, Hypothyroidism, Chronic Kidney Disease, Depression | Yes | No | Non death |
| 62 | 2414818H | Female | Hypertension, Diabetes Mellitus, Dyslipidemia, Coronary Artery Disease (Symptomatic or Positive Testing) | Yes | No | Non death |
| 62 | 13943600D | Male | Chronic Obstructive Pulmonary Disease (COPD, emphysema) | Yes | No | Non death |
| 59 | 89500170K | Male | Diabetes Mellitus, Hypothyroidism, Chronic Kidney Disease, Epilepsy | Yes | No | Non death |
| 48 | 14061864E | Female | Hypertension, Diabetes Mellitus, Heart Failure, Smoker, Obstructive Sleep Apnea | Yes | No | Non death |
| 59 | 3341505K | Male | Hypertension, Diabetes Mellitus, Dyslipidemia, Heart Failure, Asthma, Obstructive Sleep Apnea; Depression | Yes | No | Non death |
| 41 | 13853438G | Male | Hypertension, Asthma | Yes | No | Non death |
| 81 | 14086295D | Male | Hypertension, Diabetes Mellitus, Chronic Kidney Disease | Yes | No | Non death |
| 62 | 3336284C | Female | Hypertension, Diabetes Mellitus, Dyslipidemia, Hypothyroidism, Osteoarthritis , Smoker | Yes | No | Non death |
| 57 | 13914281H | Female | Hypertension, Dyslipidemia, Chronic Obstructive Pulmonary Disease (COPD, emphysema), Asthma | Yes | No | Non death |
| 69 | 3322105A | Male | Hypertension, Diabetes Mellitus, Previous Stroke or TIA | Yes | No | Non death |
| 35 | 14128478G | Female | Systemic Lupus | Yes | No | Non death |
| 62 | 13929777I | Male | Hypertension, Diabetes Mellitus, Rheumatoid Arthritis | Yes | No | Non death |
| 41 | 13879607K | Male | Hypertension, Diabetes Mellitus, Dyslipidemia, Schizophrenia, Obesity, Obstructive Sleep Apnea | Yes | No | Non death |
| 34 | 14143737J | Female | Dyslipidemia, Hypothyroidism, Obesity | Yes | No | Non death |
| 26 | 14069272J | Female | Doença de Still no adulto | Yes | No | Non death |
| 62 | 13448389D | Female | Hypertension, Diabetes Mellitus, Dyslipidemia, Hypothyroidism | Yes | No | Non death |
| 52 | 14140200F | Female | Hypertension, Diabetes Mellitus, Dyslipidemia, Obesity | Yes | No | Non death |
| 71 | 5264822D | Male | Hypertension, Diabetes Mellitus, Dyslipidemia, Chronic Kidney Disease, Coronary Artery Disease (Symptomatic or Positive Testing), Heart Failure, Cancer | Yes | No | Non death |
| 65 | 2350964G | Female | Hypothyroidism, Depression, Panhipopituitarismo cirurgico (macroadenoma) | Yes | No | Non death |
| 88 | 14101929D | Female | Hypertension, Diabetes Mellitus, Hypothyroidism, Chronic Kidney Disease, Previous Stroke or TIA | Yes | No | Non death |
| 68 | 55754170A | Female | Hypertension, Diabetes Mellitus, Chronic Kidney Disease, Heart Failure, Chronic Obstructive Pulmonary Disease (COPD, emphysema), Osteoporosis, Obesity | Yes | No | Non death |
| 69 | 14058414G | Female | Hypertension, Dyslipidemia, Coronary Artery Disease (Symptomatic or Positive Testing), Cancer | Yes | No | Non death |
| 53 | 14128423E | Female | Asthma, Fibromyalgia , Obesity, Gastroesophageal Reflux | Yes | No | Non death |
| 62 | 3379269I | Female | Hypertension, Diabetes Mellitus, Dyslipidemia, Hypothyroidism, Coronary Artery Disease (Symptomatic or Positive Testing), Heart Failure, Osteoarthritis , Obesity | Yes | No | Non death |
| 47 | 3205129F | Female | Dyslipidemia, Hypothyroidism | Yes | No | Non death |
| 67 | 14041192E | Female | Hypertension, Diabetes Mellitus, Dyslipidemia, Chronic Kidney Disease | Yes | No | Non death |
| 55 | 3319932K | Female | Hypertension, Diabetes Mellitus, Dyslipidemia, Hypothyroidism | Yes | No | Non death |
| 73 | 13624938C | Female | Hypertension, Dyslipidemia, Hypothyroidism, Osteoarthritis | Yes | No | Non death |
| 80 | 3320114I | Male | Hypertension, Diabetes Mellitus, Dyslipidemia, Hypothyroidism, Coronary Artery Disease (Symptomatic or Positive Testing) | Yes | No | Non death |
| 65 | 13514709J | Female | Hypertension, Diabetes Mellitus, Dyslipidemia, Hypothyroidism, Osteoarthritis , Previous Stroke or TIA | Yes | No | Non death |
| 43 | 14031430H | Male | Hypertension, Sarcoidosis | Yes | No | Non death |
| 69 | 44206757F | Male | Dyslipidemia, Osteoporosis, Systemic Sclerosis | Yes | No | Non death |
| 51 | 14162526D | Female | Hypertension, Diabetes Mellitus, Chronic Kidney Disease, Coronary Artery Disease (Symptomatic or Positive Testing), Heart Failure | Yes | No | Non death |
| 61 | 14084267J | Female | Hypertension, Diabetes Mellitus, Dyslipidemia, Hypothyroidism, Chronic Kidney Disease, Osteoporosis | Yes | No | Non death |
| 59 | 14145333I | Male | Diabetes Mellitus, Chronic Kidney Disease, Heart Failure, Atrial Fibrillation | Yes | No | Non death |
| 59 | 13809278C | Female | Hypertension, Diabetes Mellitus, Heart Failure | Yes | No | Non death |
| 46 | 90659240F | Female | Hypertension, Obstructive Sleep Apnea; Pulmonary Embolism | Yes | No | Non death |
| 39 | 13962276H | Female | Hypothyroidism, Asthma | Yes | No | Non death |
| 79 | 44115994F | Female | Hypertension, Dyslipidemia, Chronic Kidney Disease | Yes | No | Non death |
| 87 | 13807306J | Female | Hypertension, Dyslipidemia, Osteoarthritis , Osteoporosis, Smoker | Yes | No | Non death |
| 69 | 14147711B | Female | Hypertension, Heart Failure, Chronic Obstructive Pulmonary Disease (COPD, emphysema) | Yes | No | Non death |
| 57 | 3020042E | Female | Hypertension, Diabetes Mellitus, Dyslipidemia, Smoker | Yes | No | Non death |
| 72 | 13655001J | Female | Hypertension, Diabetes Mellitus, Dyslipidemia, Chronic Kidney Disease | Yes | No | Non death |
| 73 | 13442491B | Female | Hypertension, Dyslipidemia, Rheumatoid Arthritis, Smoker, Cancer | Yes | No | Non death |
| 44 | 3253016G | Male | Diabetes Mellitus, impotencia sPreviousual, Glaucoma, Chronic Venous Insufficiency | Yes | No | Non death |
| 78 | 13999717G | Female | Hypertension, Osteoarthritis , Osteoporosis, Polymyalgia Rheumatica , rosácea | Yes | No | Non death |
| 77 | 2870725A | Female | Hypertension, Hypothyroidism, Anemia (with previous prescription of medical treatment or transfusion), Atrial Fibrillation, Schizophrenia,Peripheral Artery Disease, Pulmonary Embolism | Yes | No | Non death |
| 54 | 14118112E | Male | Hypertension, Diabetes Mellitus, Dyslipidemia, Chronic Kidney Disease, Heart Failure, Lung Restrictive Disease | Yes | No | Non death |
| 58 | 14138342I | Female | Hypertension, Diabetes Mellitus, Chronic Kidney Disease, Heart Failure, Previous Smoker, Previous Alcohol Use Disorder, Benign Prostatic Hyperplasia sintomática. | Yes | No | Non death |
| 66 | 14115734J | Female | Hypertension, Diabetes Mellitus, Coronary Artery Disease (Symptomatic or Positive Testing), Smoker, Estenose arteria vertebral E | Yes | No | Non death |
| 57 | 2797767B | Male | Hypertension, Diabetes Mellitus, Dyslipidemia, Chronic Kidney Disease, Previous Stroke or TIA, Previous Smoker, Chronic Low Back Pain | Yes | No | Non death |
| 56 | 3370896B | Female | Rheumatoid Arthritis, Osteopenia, Peripheral Neuropathy. | Yes | No | Non death |
| 72 | 55735686A | Female | Hypertension, Diabetes Mellitus, Dyslipidemia, Osteoporosis, Fibromyalgia , Atrial Fibrillation, Overweight, Cataract | Yes | No | Non death |
| 58 | 2927088G | Female | Heart Failure, Myocarditis | Yes | No | Non death |
| 46 | 3163304A | Female | Hypertension, Diabetes Mellitus, Dyslipidemia, Hypothyroidism, Nephrotic Syndrome, Glaucoma, Cataract | Yes | No | Non death |
| 54 | 13597137H | Female | Hypertension, Hypothyroidism, Osteoarthritis , Doença ulcerosa péptica, Obesity, Hepatic Steatosis, Low Member Livedoid Vasculopathy | Yes | No | Non death |
| 30 | 14099372J | Female | Systemic Lupus, Anemia (with previous prescription of medical treatment or transfusion) | Yes | No | Non death |
| 80 | 13786995A | Female | Hypertension, Diabetes Mellitus, Dyslipidemia, Hypothyroidism, Heart Failure, Previous Stroke or TIA, Cataract, Osteopenia, Low Member Varices | Yes | No | Non death |
| 54 | 90526240B | Female | Hypertension, Diabetes Mellitus, Dyslipidemia, Hypothyroidism, Chronic Kidney Disease, Heart Failure, Hernia de disco | Yes | No | Non death |
| 73 | 3075764B | Male | Hypertension, Diabetes Mellitus, Hypothyroidism, Coronary Artery Disease (Symptomatic or Positive Testing), Osteoporosis, Bullous Pemphigus, Previous Alcohol Use Disorder | Yes | No | Non death |
| 58 | 3249807H | Female | Hypertension, Dyslipidemia, Hypothyroidism, Systemic Lupus, Depression | Yes | No | Non death |
| 48 | 2803673H | Male | Diabetes Mellitus, Cirrhosis por HCV | Yes | No | Non death |
| 40 | 90839213J | Female | Hypertension, Rheumatoid Arthritis | Yes | No | Non death |
| 61 | 13914537E | Female | Hypertension, Osteoarthritis , Rheumatoid Arthritis | Yes | No | Non death |
| 61 | 13473775B | Female | Hypertension, Diabetes Mellitus, Dyslipidemia, Previous Stroke or TIA, Smoker, Depression | Yes | No | Non death |
| 66 | 13457271G | Female | Hypertension, Diabetes Mellitus, Chronic Kidney Disease, Osteoarthritis , Rheumatoid Arthritis, Depression | Yes | No | Non death |
| 75 | 13921435I | Male | Hypertension, Dyslipidemia, Chronic Kidney Disease, Heart Failure, Osteoarthritis , Epilepsy | Yes | No | Non death |
| 22 | 14176276H | Female | Systemic Lupus | Yes | No | Non death |
| 71 | 3350012H | Female | Hypertension, Diabetes Mellitus, Dyslipidemia, Hypothyroidism, Chronic Kidney Disease, Coronary Artery Disease (Symptomatic or Positive Testing), Heart Failure, Osteoarthritis , Obesity, Depression | Yes | No | Non death |
| 68 | 2303822G | Female | Hypertension, Diabetes Mellitus, Dyslipidemia, Hypothyroidism, Chronic Kidney Disease, Cancer, Obesity | Yes | No | Non death |
| 44 | 3344549B | Female | Rheumatoid Arthritis, Depression | Yes | No | Non death |
| 37 | 13737108E | Female | Systemic Lupus | Yes | No | Non death |
| 57 | 13947708E | Female | Hypertension, Diabetes Mellitus, Hypothyroidism, Coronary Artery Disease (Symptomatic or Positive Testing) | Yes | No | Non death |
| 33 | 14156084E | Male | Smoker, Ankylosing Spondylitis | Yes | No | Non death |
| 65 | 13744749J | Female | Hypertension, Diabetes Mellitus, Dyslipidemia, Heart Failure, Osteoarthritis , Systemic Lupus, Depression | Yes | No | Non death |
| 55 | 13985780H | Female | Hypertension, Dyslipidemia, Hypothyroidism, Osteoarthritis | Yes | No | Non death |
| 60 | 3153971I | Male | Hypertension, Diabetes Mellitus, Smoker, Benign Prostatic Hyperplasia | Yes | No | Non death |
| 65 | 14003136K | Male | Diabetes Mellitus, Dyslipidemia, Hypothyroidism | Yes | No | Non death |
| 39 | 3273847H | Female | Asthma, Systemic Lupus, Allergic Rhinitis | Yes | No | Non death |
| 68 | 13929591F | Male | Hypertension, Diabetes Mellitus, Hypothyroidism, Depression | Yes | No | Non death |
| 56 | 3353541I | Female | Hypertension, Diabetes Mellitus, Dyslipidemia, Rheumatoid Arthritis, Obesity, Depression | Yes | No | Non death |
| 60 | 13986545G | Female | Hypertension, Heart Failure, Osteoporosis, Hyperthyroidism | Yes | No | Non death |
| 42 | 3289924J | Female | Dyslipidemia, Osteoarthritis , Rheumatoid Arthritis, Depression | Yes | No | Non death |
| 61 | 13500237E | Female | Hypertension, Diabetes Mellitus, Dyslipidemia, Coronary Artery Disease (Symptomatic or Positive Testing), Asthma, Osteoarthritis , Obesity, Depression | Yes | No | Non death |
| 55 | 90592757E | Male | Hypertension, Dyslipidemia, Chronic Kidney Disease, Coronary Artery Disease (Symptomatic or Positive Testing), Heart Failure, Obesity | Yes | No | Non death |
| 35 | 14150086K | Female | Hypothyroidism, Systemic Lupus, Depression | Yes | No | Non death |
| 58 | 2805569J | Female | Hypertension, Diabetes Mellitus, Dyslipidemia, Fibromyalgia | Yes | No | Non death |
| 65 | 2645383H | Male | Hypertension, Diabetes Mellitus, Dyslipidemia, Chronic Kidney Disease, Alcohol Use Disorder | Yes | No | Non death |
| 47 | 3085778K | Female | Hypertension, Diabetes Mellitus, Dyslipidemia, Hypothyroidism, Obesity, Depression | Yes | No | Non death |
| 67 | 55405746D | Male | Hypertension, Diabetes Mellitus, Dyslipidemia, Chronic Kidney Disease, Coronary Artery Disease (Symptomatic or Positive Testing), Smoker | Yes | No | Non death |
| 70 | 7047745E | Female | Osteoarthritis , Osteoporosis, Rheumatoid Arthritis, Systemic Lupus | Yes | No | Non death |
| 63 | 13805238B | Female | Hypertension, Diabetes Mellitus, Dyslipidemia, Coronary Artery Disease (Symptomatic or Positive Testing) | Yes | No | Non death |
| 57 | 13504818J | Male | Osteoporosis, Doença de Still | Yes | No | Non death |
| 41 | 13995629D | Female | Hypertension, Systemic Lupus, Obesity | Yes | No | Non death |
| 81 | 13606784B | Male | Hypertension, Diabetes Mellitus, Dyslipidemia, Coronary Artery Disease (Symptomatic or Positive Testing), Chronic Obstructive Pulmonary Disease (COPD, emphysema), Benign Prostatic Hyperplasia | Yes | No | Non death |
| 55 | 55527975C | Male | Hypertension, Coronary Artery Disease (Symptomatic or Positive Testing), Cancer, Pulmonary Embolism | Yes | No | Non death |
| 49 | 13580350I | Female | Hypertension, Diabetes Mellitus, Hypothyroidism, Osteoarthritis , Systemic Lupus | Yes | No | Non death |
| 77 | 13662263B | Female | Hypertension, Dyslipidemia, Hypothyroidism, Coronary Artery Disease (Symptomatic or Positive Testing), Cancer | Yes | No | Non death |
| 55 | 13856323D | Female | Hypertension, Dyslipidemia, Hypothyroidism, Heart Failure, Chronic Obstructive Pulmonary Disease (COPD, emphysema), Osteoarthritis , Obesity, Obstructive Sleep Apnea | Yes | No | Non death |
| 74 | 5186192G | Female | Hypertension, Diabetes Mellitus, Dyslipidemia, Hypothyroidism, Coronary Artery Disease (Symptomatic or Positive Testing), Osteoarthritis , Pneumonite de hipersensibilidade | Yes | No | Non death |
| 68 | 13482248E | Female | Hypertension, Diabetes Mellitus, Dyslipidemia, Hypothyroidism, Asthma, Cancer, Obstructive Sleep Apnea | Yes | No | Non death |
| 77 | 13936377A | Female | Hypertension, Diabetes Mellitus, Dyslipidemia, Osteoarthritis , Depression, Fibromyalgia | Yes | No | Non death |
| 46 | 3166932J | Female | Diabetes Mellitus, Hypothyroidism, Vitiligo, Pernicious Anemia | Yes | No | Non death |
| 72 | 89501786F | Female | Hypertension, Diabetes Mellitus, Dyslipidemia, Chronic Kidney Disease, Heart Failure, Osteoporosis, NASH, Gastroesophageal Reflux | Yes | No | Non death |
| 59 | 13636001F | Male | Hypertension, Obesity, Pulmonary Embolism | Yes | No | Non death |
| 76 | 13824887A | Male | Hypertension, Dyslipidemia, Hypothyroidism, Chronic Kidney Disease, Previous Stroke or TIA, Dementia, Deep Venous Thrombosis, Pulmonary Embolism | Yes | No | Non death |
| 62 | 14119309J | Female | Hypertension, Hypothyroidism, Sjögren Syndrome | Yes | No | Non death |
| 74 | 14127483C | Female | Hypertension, Dyslipidemia, Cancer, Generalized Anxiety Disorder | Yes | No | Non death |
| 57 | 13954680B | Female | Hypertension, Diabetes Mellitus, Coronary Artery Disease (Symptomatic or Positive Testing), Heart Failure, Osteoarthritis , Smoker, Depression | Yes | No | Non death |
| 64 | 13714938B | Female | Hypertension, Diabetes Mellitus, Chronic Kidney Disease, Coronary Artery Disease (Symptomatic or Positive Testing), Osteoporosis, Cancer, Obesity | Yes | No | Non death |
| 70 | 13915011J | Male | Hypertension, Diabetes Mellitus, Chronic Kidney Disease, Heart Failure, Chronic Obstructive Pulmonary Disease (COPD, emphysema), Obesity | Yes | No | Non death |
| 22 | 13776766A | Female | Systemic Lupus, Cancer, Epilepsy | Yes | No | Non death |
| 33 | 13670205k | Female | Rheumatoid Arthritis | Yes | No | Non death |
| 55 | 14152520E | Male | Hypertension, Diabetes Mellitus, Alcohol induced Cirrhosis, Obesity, Depression | Yes | No | Non death |
| 54 | 55747797J | Female | Hypertension, Diabetes Mellitus, Hypothyroidism, Chronic Kidney Disease, Heart Failure | Yes | No | Non death |
| 42 | 13661890J | Male | Hypertension, Diabetes Mellitus, Dyslipidemia, Obesity | Yes | No | Non death |
| 64 | 13627259I | Female | Hypertension, Dyslipidemia, Osteoarthritis , Obesity | Yes | No | Non death |
| 35 | 14012781I | Female | Systemic Lupus | Yes | No | Non death |
| 39 | 13899504F | Female | Hypertension, Diabetes Mellitus, Miastenia gravis | Yes | No | Non death |
| 82 | 3388185J | Female | Hypertension, Diabetes Mellitus, Dyslipidemia, Hypothyroidism, Chronic Kidney Disease, Heart Failure, Chronic Obstructive Pulmonary Disease (COPD, emphysema) | Yes | No | Non death |
| 68 | 3159283B | Female | Hypertension, Diabetes Mellitus, Dyslipidemia, Osteoporosis, Rheumatoid Arthritis | Yes | No | Non death |
| 57 | 4083924B | Female | Hypertension, Graves Disease | Yes | No | Non death |
| 67 | 3162115J | Female | Diabetes Mellitus, Rheumatoid Arthritis, Síndrome de Sjogren, Fibromyalgia | Yes | No | Non death |
| 60 | 14121174C | Female | Hypertension, Diabetes Mellitus, Coronary Artery Disease (Symptomatic or Positive Testing) | Yes | No | Non death |
| 65 | 13745268I | Male | Hypertension, Dyslipidemia, Cancer, Polymyositis | Yes | No | Non death |
| 54 | 2974029I | Female | Hypertension, Rheumatoid Arthritis | Yes | No | Non death |
| 37 | 14130050E | Male | Hypertension, Diabetes Mellitus | Yes | No | Non death |
| 90 | 14072762K | Female | Hypertension, Diabetes Mellitus, Chronic Kidney Disease, Osteoarthritis , Osteoporosis, Previous Stroke or TIA | Yes | No | Non death |
| 35 | 13437941K | Female | Diabetes Mellitus | Yes | No | Non death |
| 67 | 14020110D | Female | Osteoporosis, Hyperthyroidism | Yes | No | Non death |
| 76 | 13668678C | Male | Hypertension, Diabetes Mellitus, Dyslipidemia, Chronic Kidney Disease, Coronary Artery Disease (Symptomatic or Positive Testing), Smoker, Aortic Aneurysm | Yes | No | Non death |
| 54 | 13489648I | Female | Hypertension, Diabetes Mellitus, Dyslipidemia | Yes | No | Non death |
| 41 | 14116872B | Female | Hypertension, Smoker, Hyperthyroidism | Yes | No | Non death |
| 79 | 13508771E | Female | Hypertension, Diabetes Mellitus, Dyslipidemia, Coronary Artery Disease (Symptomatic or Positive Testing), Heart Failure, Cancer, Obesity | Yes | Yes | Non death |
| 65 | 3087693D | Female | Infflamtoty Bowel Syndromel, Glaucoma | Yes | No | Non death |
| 64 | 14044866K | Male | Hypertension, Diabetes Mellitus, Chronic Kidney Disease, Cancer, Flutter atrial, Previous Smoker, Previous Alcohol Use Disorder | Yes | No | Non death |
| 58 | 13876932J | Female | Hypertension, Dyslipidemia, Heart Failure, Anemia (with previous prescription of medical treatment or transfusion), Chagas Disease, Obesity, Pre Diabetes, Previous Smoker | Yes | No | Non death |
| 55 | 2870889E | Female | Hypertension, Osteoarthritis , Hyperthyroidism, Obesity, Functional Dyspepsia., Fibromyalgia | Yes | No | Non death |
| 39 | 13448344J | Female | Dyslipidemia, Hypothyroidism, Depression, Obesity, Endometriosis | Yes | No | Non death |
| 29 | 90898937G | Female | Migraine, Pulmonary Embolism | Yes | No | Non death |
| 60 | 14150138I | Female | Hypertension, Diabetes Mellitus, Dyslipidemia, Hypothyroidism, Obesity , Functional Dyspepsia. | Yes | No | Non death |
| 66 | 13915263G | Male | Hypertension, Diabetes Mellitus, Dyslipidemia, Chronic Kidney Disease, Coronary Artery Disease (Symptomatic or Positive Testing), Heart Failure, Anemia (with previous prescription of medical treatment or transfusion), Cataract, Depression | Yes | No | Non death |
| 43 | 3238302K | Male | Hypertension, Diabetes Mellitus, Dyslipidemia, Epilepsy | Yes | No | Non death |
| 60 | 13879810G | Female | Hypertension, Diabetes Mellitus, Coronary Artery Disease (Symptomatic or Positive Testing), Systemic Sclerosis sistemica, calcinose, Obstructive Sleep Apnea | Yes | No | Non death |
| 64 | 13676242E | Female | Hypertension, Diabetes Mellitus, Heart Failure, Sarcoidosis, Syringomyelia, Gastroesophageal Reflux, Gallstone, Depression, Obstructive Sleep Apnea | Yes | No | Non death |
| 43 | 13691437H | Female | Asthma, Systemic Lupus, Lues secundário | Yes | No | Non death |
| 40 | 13940395J | Female | Dyslipidemia, Systemic Lupus, Sjögren Syndrome, Pre- diabetes, Fibromyalgia , Migraine, Overweight | Yes | No | Non death |
| 66 | 13909328G | Female | Hypertension, Diabetes Mellitus, Osteoarthritis , Cancer, Erosive Gastritis, Diverticulosis | Yes | No | Non death |
| 63 | 2934094F | Female | Hypertension, Diabetes Mellitus, Heart Failure, Osteoarthritis , Tension Type Headache, Psichiatric Disorder | Yes | No | Non death |
| 48 | 13470453D | Male | Hypertension, Diabetes Mellitus, Obesity, Obstructive Sleep Apnea | Yes | No | Non death |
| 45 | 2106312G | Male | Hypertension, Dyslipidemia, Caustic Esophagus Stenosis, Amaurosis on Right Eye | Yes | No | Non death |
| 29 | 14174830A | Female | Chronic Kidney Disease, Heart Failure, Depression | Yes | No | Non death |
| 48 | 14126372E | Female | Asthma, Anemia (with previous prescription of medical treatment or transfusion), | Yes | No | Non death |
| 69 | 3137775E | Female | Hypertension, Diabetes Mellitus, Dyslipidemia, Hypothyroidism | Yes | No | Non death |
| 62 | 3109204K | Female | Hypertension, Diabetes Mellitus, Dyslipidemia | Yes | No | Non death |
| 67 | 14015016E | Male | Osteoporosis, Anemia (with previous prescription of medical treatment or transfusion), Relapsing Retroauricular Abscess, Chronic Pulmonary Embolism | Yes | No | Non death |
| 85 | 3343491D | Male | Hypertension, Dementia | Yes | No | Non death |
| 72 | 77128830I | Male | Hypertension, Diabetes Mellitus, Chronic Kidney Disease, Heart Failure, Peripheral Artery Disease, | Yes | Yes | Non death |
| 63 | 14175075K | Male | Diabetes Mellitus, Dyslipidemia, Chronic Obstructive Pulmonary Disease (COPD, emphysema), Smoker, Schizophrenia | Yes | No | Non death |
| 69 | 14048805J | Male | Hypertension, Diabetes Mellitus, Dyslipidemia, Coronary Artery Disease (Symptomatic or Positive Testing), Smoker | Yes | No | Non death |
| 51 | 2448848E | Female | Osteoporosis, Rheumatoid Arthritis, Smoker, Cancer | Yes | No | Non death |
| 71 | 90988243B | Female | Hypertension, Coronary Artery Disease (Symptomatic or Positive Testing), Chronic Obstructive Pulmonary Disease (COPD, emphysema) | Yes | No | Non death |
| 55 | 14055558B | Male | Hypertension, Diabetes Mellitus, Dyslipidemia | Yes | No | Non death |
| 87 | 2366673C | Male | Hypertension, Diabetes Mellitus, Heart Failure, Osteoporosis | Yes | No | Non death |
| 64 | 3240981I | Male | Hypertension, Diabetes Mellitus, Dyslipidemia, Heart Failure, Cancer | Yes | No | Non death |
| 68 | 2452513K | Female | Hypertension, Diabetes Mellitus, Dyslipidemia, Hypothyroidism, Coronary Artery Disease (Symptomatic or Positive Testing), Osteoarthritis , Osteoporosis | Yes | No | Non death |
| 56 | 14099137C | Female | Hypertension, Diabetes Mellitus, Dyslipidemia, Hypothyroidism, Coronary Artery Disease (Symptomatic or Positive Testing) | Yes | No | Non death |
| 60 | 13654265G | Male | Hypertension, Diabetes Mellitus, Dyslipidemia, Thromboangiitis Obliterans | Yes | No | Non death |
| 69 | 5283032D | Male | Hypertension, Diabetes Mellitus, Coronary Artery Disease (Symptomatic or Positive Testing), Heart Failure, Peripheral Artery Disease | Yes | No | Non death |
| 56 | 13485271J | Female | Hypertension, Diabetes Mellitus, Dyslipidemia, Coronary Artery Disease (Symptomatic or Positive Testing) | Yes | No | Non death |
| 58 | 13912220D | Female | Hypertension, Rheumatoid Arthritis | Yes | No | Non death |
| 40 | 79003310H | Female | Asthma, Anemia (with previous prescription of medical treatment or transfusion) | Yes | No | Non death |
| 85 | 14053076F | Female | Diabetes Mellitus, Hypothyroidism, Chronic Kidney Disease, Exocrine ancreatic Insuffciency | Yes | No | Non death |
| 63 | 90894753K | Male | Heart Failure | Yes | No | Non death |
| 27 | 13993629C | Female | Takayasu Arthritis | Yes | No | Non death |
| 45 | 2895163K | Female | Hypertension, Diabetes Mellitus, Dyslipidemia | Yes | No | Non death |
| 84 | 3118454I | Female | Hypertension, Diabetes Mellitus, Dyslipidemia, Hypothyroidism, Chronic Kidney Disease | Yes | No | Non death |
| 67 | 3227073A | Male | Hypertension, Diabetes Mellitus | Yes | No | Non death |
| 51 | 14022977C | Female | Hypertension, Heart Failure, Chronic Obstructive Pulmonary Disease (COPD, emphysema), Smoker | Yes | No | Non death |
| 51 | 13947714F | Female | Dyslipidemia, Systemic Lupus | Yes | No | Non death |
| 79 | 14106414G | Female | Hypertension, Diabetes Mellitus, Heart Failure, Peripheral Artery Disease | Yes | No | Non death |
| 39 | 3142671J | Female | Hypertension, Systemic Lupus | Yes | No | Non death |
| 59 | 13819628G | Female | Hypertension, Diabetes Mellitus, Dyslipidemia, Osteoarthritis , Smoker | Yes | No | Non death |
| 56 | 13522700A | Female | Diabetes Mellitus, Dyslipidemia, Osteoarthritis | Yes | No | Non death |
| 67 | 13741101C | Female | Hypertension, Diabetes Mellitus, Dyslipidemia, Hypothyroidism, Coronary Artery Disease (Symptomatic or Positive Testing) | Yes | No | Non death |
| 39 | 13947723D | Male | Hypertension, Dyslipidemia, Chronic Kidney Disease, Heart Failure | Yes | No | Non death |
| 34 | 2490348B | Female | Diabetes Mellitus | Yes | No | Non death |
| 25 | 90567663C | Female | Hemostasis Disorder | Yes | No | Non death |
| 19 | 14177770K | Female | Asthma, Cancer, Anemia (with previous prescription of medical treatment or transfusion) | Yes | No | Non death |
| 77 | 13533208B | Female | Hypertension, Diabetes Mellitus, Dyslipidemia, Hypothyroidism, Chronic Kidney Disease, Chronic Obstructive Pulmonary Disease (COPD, emphysema), Osteoarthritis , Osteoporosis, Anemia (with previous prescription of medical treatment or transfusion) | Yes | No | Non death |
| 50 | 90469122K | Female | Epilepsy | Yes | No | Non death |
| 30 | 14169823D | Female | Diabetes Mellitus, Chronic Kidney Disease, Heart Failure | Yes | No | Non death |
| 51 | 14072935I | Female | Hypothyroidism, Rheumatoid Arthritis | Yes | No | Non death |
